# Impact of relational continuity of primary care in aged care: a systematic review

**DOI:** 10.1186/s12877-022-03131-2

**Published:** 2022-07-14

**Authors:** Suzanne M. Dyer, Jenni Suen, Helena Williams, Maria C. Inacio, Gillian Harvey, David Roder, Steve Wesselingh, Andrew Kellie, Maria Crotty, Gillian E. Caughey

**Affiliations:** 1 Flinders Health and Medical Research Institute, Flinders University, Adelaide, Australia; 2 Silver Chain Group, Adelaide, Australia; 3 Registry of Senior Australians, South Australian Health and Medical Research Institute, Adelaide, Australia; 4 Allied Health and Human Performance, University of South Australia, Adelaide, Australia; 5 College of Nursing and Health Sciences, Flinders University, Adelaide, Australia; 6 South Australian Health and Medical Research Institute, Adelaide, Australia; 7 East Adelaide Healthcare, Adelaide, Australia

**Keywords:** Primary care, Aged care, Long-term care, Systematic review, Hospitalisation, General practitioner

## Abstract

**Background:**

Greater continuity of care has been associated with lower hospital admissions and patient mortality. This systematic review aims to examine the impact of relational continuity between primary care professionals and older people receiving aged care services, in residential or home care settings, on health care resource use and person-centred outcomes.

**Methods:**

Systematic review of five databases, four trial registries and three grey literature sources to October 2020. Included studies (a) aimed to increase relational continuity with a primary care professional, (b) focused on older people receiving aged care services (c) included a comparator and (d) reported outcomes of health care resource use, quality of life, activities of daily living, mortality, falls or satisfaction. Cochrane Collaboration or Joanna Briggs Institute criteria were used to assess risk of bias and GRADE criteria to rate confidence in evidence and conclusions.

**Results:**

Heterogeneity in study cohorts, settings and outcome measurement in the five included studies (one randomised) precluded meta-analysis. None examined relational continuity exclusively with non-physician providers. Higher relational continuity with a primary care physician probably reduces hospital admissions (moderate certainty evidence; high versus low continuity hazard ratio (HR) 0.94; 95% confidence interval (CI) 0.92–0.96, *n* = 178,686; incidence rate ratio (IRR) 0.99, 95%CI 0.76–1.27, *n* = 246) and emergency department (ED) presentations (moderate certainty evidence; high versus low continuity HR 0.90, 95%CI 0.89–0.92, *n* = 178,686; IRR 0.91, 95%CI 0.72–1.15, *n* = 246) for older community-dwelling aged care recipients. The benefit of providing on-site primary care for relational continuity in residential settings is uncertain (low certainty evidence, 2 studies, *n* = 2,468 plus 15 care homes); whilst there are probably lower hospitalisations and may be fewer ED presentations, there may also be an increase in reported mortality and falls. The benefit of general practitioners’ visits during hospital admission is uncertain (very low certainty evidence, 1 study, *n* = 335).

**Conclusion:**

Greater relational continuity with a primary care physician probably reduces hospitalisations and ED presentations for community-dwelling aged care recipients, thus policy initiatives that increase continuity may have cost offsets. Further studies of approaches to increase relational continuity of primary care within aged care, particularly in residential settings, are needed.

**Review registration:**

CRD42021215698.

**Supplementary Information:**

The online version contains supplementary material available at 10.1186/s12877-022-03131-2.

## Introduction

The provision of good quality primary care is considered pivotal to providing “an efficient, equitable and effective health system” [1, 2]. Primary care is the initial contact point in the healthcare system for many people, thus primary care professionals play a key role in disease prevention, treatment, and rehabilitation, predominantly for people living in the community. Accessible and effective primary care is also critical for those living with complex and chronic conditions and provides an important gateway to necessary specialist services. The quality of primary care therefore becomes increasingly important as people age and disease complexity and multimorbidity becomes more prevalent [2].

Organisation for Economic Co-operation and Development data indicates that approximately 10–20% of people over 65 years of age receive aged care services (i.e., aged care recipients, or older people receiving long-term care), with the proportion of care delivered in institutional versus home care settings varying between nations [3]. Older people, particularly aged care recipients, are at high risk of emergency department (ED) presentations and hospital admissions due in part to frailty, falls, the high prevalence of chronic conditions and multimorbidity [4–7]. These events are costly and lead to poor health outcomes including further decline in function and quality of life. Thus, there is increasing emphasis internationally on finding strategies to reduce potentially preventable hospitalisations and ED presentations [8–11]. The provision of higher-quality primary care could reduce some of these hospital presentations, other health care wastage and improve aged care recipients’ health and wellbeing [10, 12, 13]. A previous systematic review has found an association of increased provider continuity with decreased healthcare utilisation and improved patient satisfaction [14]. Increasing care continuity has been a focus of policy agencies to improve care and reduce hospitalisations for older people generally [10, 12]. Higher continuity of care with a general practitioner (GP) has been associated with a lower risk of hospital admission for ambulatory care sensitive conditions in older people in the general population [15, 16]. In Norway, higher relational continuity with a GP is associated with decreased use of out-of-hours services, acute hospitalisations and mortality, an effect that was greater with a longer relationship duration [17]. In a Canadian study, 53% of the participants had some difficulty in performing instrumental activities of daily living and 20% had difficulty with basic activities of daily living so may be likely to be receiving some aged or social care services [16]. This association of lower hospitalisations with higher continuity of care is therefore likely to be applicable to older adults receiving aged care services. Higher continuity of care with physicians has also been associated with a lower likelihood of hospital admissions and ED presentations for people with conditions that are highly prevalent in aged care recipients, including diabetes, dementia or those with multiple chronic medical conditions [18–22].

Systematic reviews have shown greater continuity is associated with lower all-cause mortality, when continuity specifically in primary care or in primary and secondary care is considered [23, 24]. Higher relational continuity of care among older people has also been reported to have a positive impact on a range of other outcomes including fewer duplicated medications, and in people living with dementia, safer prescribing, fewer episodes of delirium and less incontinence [19, 25].

Thus, continuity with a primary care professional is considered a priority for implementation of the World Health Organisation framework on integrated people-centred health services [12]. The King’s Fund in the United Kingdom (UK) has recommended increasing continuity of care with primary care professionals to reduce hospital admissions [2, 10]. In Australia, the “continuity model” has been stated as the preferred model of care for the provision of primary care for older people by the Royal Australian College of General Practitioners (RACGPs) [26–28]. In some countries, including the UK, Norway, Denmark and the Netherlands, most people are listed with a regular general practitioner (GP) or practice as their primary healthcare provider [17].

There are three types of continuity of care: relational, management and informational continuity [29]. Relational continuity refers to the continuous relationship between a practitioner and a patient, beyond a specific episode of illness [27, 29]. Relational continuity may exist with a single practitioner, the practice (site continuity) or amongst teams with established relationships within a practice [30]. Management continuity refers to the coordination of a person’s care across the health care system, or consistency of care delivery [31]. Examples of approaches that may increase management continuity include case management, care co-ordination or system navigators. Informational continuity refers to the communication between care providers to ensure information relevant to the patient’s care travels with the patient through the health system, for example through health records [32, 33]. Relational continuity is the most referred to type of continuity in primary care and has been suggested as a mechanism for improved effectiveness [24, 27, 29]. Good relational continuity of care is expected to improve both management and informational continuity and is the type of continuity most valued by patients [27, 29, 34]. Thus, this review focuses on the evidence for the impact of relational continuity.

Recent systematic reviews have synthesised evidence for the impact of continuity of care on mortality and health care utilisation and patient satisfaction in patients of any age [14, 23, 24]. Given the significance of this topic to current policy initiatives attempting to improve outcomes for older people receiving aged care services [35], a systematic review was undertaken to examine the impact of increased or alternative models of improving relational continuity in primary care for aged care recipients, on person-centred outcomes and healthcare resource use (including hospitalisations, attendances, residential aged care admission, social service use, prescribed medications, diagnostic services etc.).

## Methods

This review was conducted according to an a priori protocol registered on PROSPERO International prospective register of systematic reviews (registration number CRD42021215698) [36]. It addresses the research question ‘What is the impact of relational continuity of primary care for older adults receiving aged care services on person-centred outcomes, health outcomes and healthcare resource use for care recipients or their carer(s) in comparison to an alternative approach?’. The review findings have been reported according to Preferred Reporting Items for Systematic Reviews and Meta-Analyses (PRISMA) guidelines [37].

### Inclusion/exclusion criteria

Included studies were those that met the following criteria: 1) aimed to examine relational continuity with a primary care professional, 2) were conducted in older adults (80% or more of participants aged 65 years or over) 3) participants received aged care services (either residential or in the community) 4) included a comparator (e.g. pre-intervention or alternative level of continuity) and 5) reported outcomes of utilisation of health care (including hospital admissions, hospital length of stay, ED presentations, readmissions, admission or use of residential care, number of health practitioner visits, social service use, prescribed medications or diagnostic and pathology testing), person-centred outcomes and health outcomes of older adults and carers (quality of life, activities of daily living, mortality, falls, or satisfaction measured on an internationally recognised, valid scale). Aged care services were defined as long-term care services provided for personal care, medical needs, and assistance in living independently to older people, whether delivered in community or residential settings. Outcomes from any economic analysis were also eligible for inclusion where studies met the criteria of reporting one of the previously listed outcomes. Any design of primary research studies with a comparator was eligible for inclusion. Systematic reviews were excluded, but their primary studies were examined for eligibility.

Exclusion criteria were 1) study designs with no comparator, 2) no primary aim to examine relational continuity of care, 3) study cohort not aged care recipients, 4) not primary care and 5) wrong outcomes. Additional file 1 provides a list of key excluded studies screened at full text which were excluded as they were studies of continuity in older people but not aged care recipients, were excluded after extensive discussion or consultation with a third reviewer, or as examples.

### Search and study selection

A comprehensive, systematic search was conducted from inception to 29^th^ of October 2020 in five databases (MEDLINE, CINAHL, PsycINFO, Cochrane Library: Cochrane Database of Systematic Reviews, International HTA database), four clinical trial registries (Cochrane Central Register of Controlled Trials, International Clinical Trials Registry Platform, ANZCTR and ClinicalTrials.gov) and three sources of grey literature (Open Grey, ProQuest Dissertations & Thesis Global and first 100 records from Google Scholar [38]). Text word and MeSH terms were used in a controlled search including terms related to aged, aged care, primary health and continuity of patient care. The complete search strategies are provided in Additional file 1. No date or language restrictions were applied to the search strategies. Reference lists of included studies and relevant systematic reviews were hand searched. Clinical experts were also consulted to identify relevant studies.

Following ‘A MeaSurement Tool to Assess systematic Reviews’ (AMSTAR) 2 criteria for high-quality reviews, two reviewers independently screened titles and abstracts of records from all database searches (79% of total records) and achieved 87.4% agreement on independent screening [39]. Consensus was achieved for discrepancies through discussion. At title and abstract screening, the agreement on the retrieval of records subsequently included in the review was 100%. A single reviewer screened the records from grey literature sources (21% of the total records) and consulted a second reviewer in cases of uncertainty. Two reviewers independently screened all full text records to determine eligibility against the inclusion criteria (independent agreement of 91%). Consensus was achieved by discussion of the remaining records.

### Data extraction

Data were extracted in duplicate by two independent reviewers into a proforma, created through discussion between two reviewers. Variables included author, publication, year, country, study design, primary care setting and provider, number of participants, continuity of care intervention characteristics, frequency of care contacts and prespecified outcomes. Data on subgroups with dementia or cognitive impairment were also extracted as the proportion of people receiving aged care services living with dementia or cognitive impairment is high and this group of people are at increased risk of hospitalisations and other adverse outcomes [35, 40–44].

### Risk of bias

Study quality was assessed for risk of bias according to study design. The Cochrane Risk of Bias tool version 2 assessing six areas for risk of bias was used for randomised controlled trials (RCTs) and the Joanna Briggs Critical Appraisal Tool was used for cohort and cross-sectional studies [45, 46]. The full appraisal criteria are provided in Additional file 1. Studies were assigned low risk of bias when the design reduced bias; unclear when information suggested bias had been reduced but insufficient information was reported; high risk of bias was assigned when bias had not been reduced. Risk of bias due to industry funding was considered.

### Data analysis and synthesis of results

A meta-analysis was considered inappropriate due to the clinical heterogeneity of the included studies in terms of the settings, cohorts, outcomes and interventions examined. Thus, a structured synthesis of the results is presented, grouping the included study findings by intervention type and setting. Hospitalisation is a key outcome and was the most common outcome reported across the included studies; thus, hospitalisation outcomes (including ED presentations) are reported in the main text and tables and other outcomes presented briefly in text and in Additional file 2. Where studies included multiple control arms, these were combined to determine a weighted average (using Microsoft Excel), for presentation of a single control outcome measure and ratio.

The overall certainty in the evidence supporting review findings for each setting/intervention category was assessed using the Grades of Recommendation, Assessment, Development, and Evaluation (GRADE) approach [47]. Independent assessment was completed by two authors. The GRADE approach is recommended by the Cochrane Collaboration to evaluate the quality of evidence supporting conclusions in systematic reviews [48]. This approach considers elements of study design, risk of bias in included studies, directness of evidence, heterogeneity, precision of results and other factors including the risk of publication bias, magnitude of effect and presence of a dose–response gradient in judging the certainty in the body of evidence. The item for risk of bias was informed by the risk of bias assessment as described. Directness of evidence considers the applicability of the patients, interventions, comparator and outcomes to the a priori research question. Heterogeneity (or inconsistency) addresses the degree of variation in effect size between the included studies. Precision of results considers the imprecision in the overall effect estimate, and thus is based upon the total sample size. The risk of publication bias can only be assessed when there are sufficient studies included to enable assessment of this item; this could not be assessed in the current review. A dose–response gradient considers whether there is a clear relation of changes in the outcome with higher levels of exposure to the intervention. Judgements were made following Cochrane, GRADE guidance and advice from the U.S.GRADE Network [49, 50]. The level of certainty in the evidence informed the communication and wording of review findings addressing the research question, based on standardized statements for conclusions of systematic reviews of interventions [51].

## Results

### Study selection

**Fig. 1:**
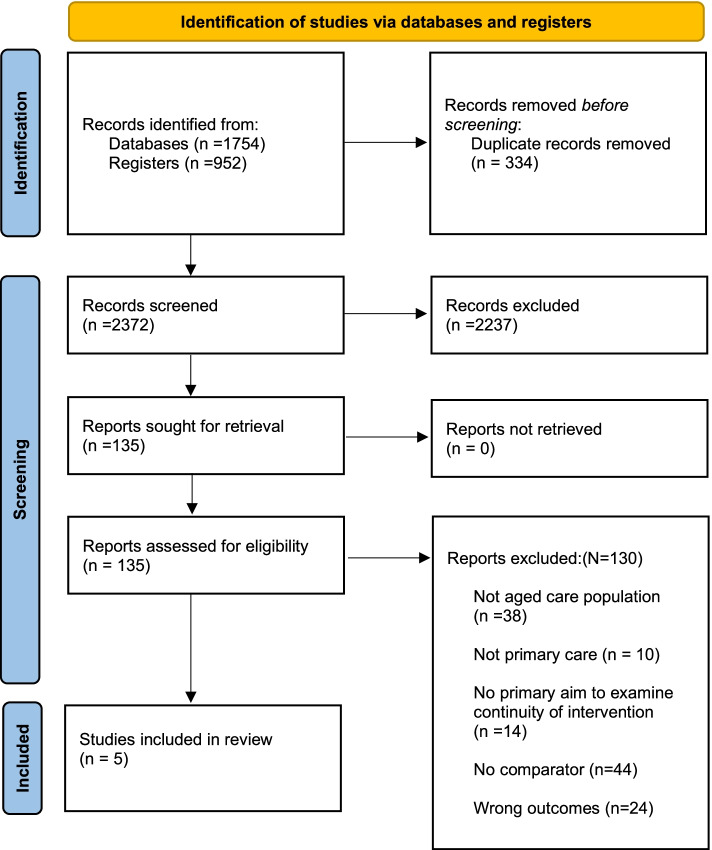
Study selection flowchart [52]

The search yielded a total of 2706 records, study selection is shown in Fig. 1. After duplicate removal, 2372 records were screened by title and abstract and 135 on full text against the inclusion and exclusion criteria. The most common exclusion reason at full text screening was the study examined an ineligible intervention. Frequently, identified studies did not indicate an aim to investigate relational continuity of care or approaches to address this. A total of five studies met the inclusion criteria and were included for review.

### Risk of bias

**Fig. 2:**
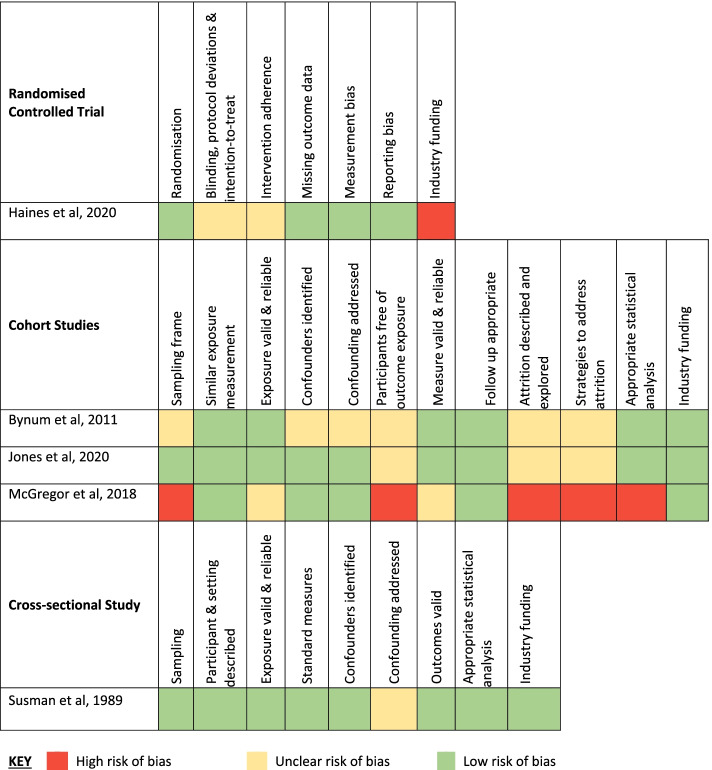
Risk of Bias in Included Studies

The five included studies were one stepped wedge cluster RCT [53], three retrospective cohort studies [54, 55] and one cross-sectional study [56]. The studies all examined different interventions or settings. Figure 2 summarises the risk of bias rating across the included studies. Overall, the included studies were predominantly at low or unclear risk of bias across the domains. Confounding, attrition, and bias from implementation fidelity were the most common aspects that contributed to the likely risk of bias amongst the included studies. Frequently, lack of information or lack of adjustment for all confounders led to an unclear risk of bias allocation. Only one small study conducted in a home care setting was considered at high risk of bias as the pre-intervention data was for a period of 21 months prior to the intervention. The patients experienced a change in their health prompting referral to the intervention program, which reduced the applicability of the historical pre-intervention data as a control period for examining the effects of the intervention [55]. In addition, the study excluded data 31 days after initial enrolment reducing completeness of data.

### Characteristics of included studies

Table 1 summarises the study characteristics. Four of the five studies captured 181,735 adults aged 65 years and over receiving aged care services either at home (178,932 people, Canada) [55, 56], in a continuing care retirement community (2468 people, United States of America (USA)) [54] or in residential care (335 people, USA) [57]. An additional Australian cluster RCT included 15 residential aged care facilities with an average of 98 beds, indicating approximately 1470 residents were likely to be contributing data [53]. Therefore, the reviewed studies captured an estimated 183,205 adults with a mean age of 74 to 85 years.

**Table 1:** Characteristics of included studies

Author, Year	Study design	Data period	Average Follow up	Country	Setting	Sample characteristics N Mean age (years) % Female Comorbidities Cognitive impairment	Continuity primary care approach & measure	Comparison	Outcome ^a^
McGregor, 2018 [55]	Retrospective cohort study	July 2008—June 2013	NR	Canada	Home Care	N: 246 Age: 85 Female: 65% Impairments: ADL: NR Cognitive: NR	Home Based Primary Care (HBPC) Program: Family physicians and NPs home visits with allied health support. A/H emergency care No CoC measure	21 months prior to HBPC, before-after ^b^	Hospitalisation ED presentation
Jones, 2020 [56]	Retrospective cohort study	Oct 2014—Sept 2016	6 months	Canada	Home Care	N: 178,686 Age: 82 Female: 61% Comorbidities (median (Q1, Q3): 3 (2,4) Impairment: ADL 41% Cognitive 59%	Continuity of care with same primary care family physician as per Bice Boxerman index (BBI), high ≥ 66.^th^ percentile (median BBI 0.88), medium 33–66 percentile (median BBI 0.73)	Low continuity ≤ 33.^rd^ percentile (median BBI 0.54)	Hospitalisation ED presentation
Bynum, 2011 [54]	Retrospective cohort study	1997–2006	1–5 years	United States	4 Continuing Care Retirement Communities	N: 2468 Age: 85 Female: 67% Cognitive: NR	On-site 3 primary care physicians and 2 part-time NPs providing all clinical care including A/H coverage on rotation. Average number of primary physicians seen	3 sites limited on-site physician hours (1.5 – 2 days). A/H coverage by physician’s practice	Hospitalisation ED presentation Primary care visits Mortality
Susman, 1989 [57]	Cross-sectional	June–Dec 1983	10.8 days	United States	Nursing home (1 site), residents transferred to hospital	*N* = 335 Age (mean): 82 Female: 72% Impairments: ADL: Y (%NR) Cognitive: NR	Continuity of care from primary physician rendering majority of routine care, while in hospital. Number of visits (1,2, ≥ 3)	Not visited by primary care physician in hospital (0 visits)	Length of stay Mortality
Haines, 2020 [53]	Stepped wedge, cluster RCT	Dec 2012—Sept 2014	54 weeks pre-and post-trial	Australia	15 private residential aged care facilities	N = NR Sites = 15 homes, mean 98 beds (SD 31) Age = NR Female = NR Impairments ADL: NR Cognitive: = NR	Standard practice: residents seen by external GPs not linked to facility staff (ideally community GP). RN undertake medication rounds and complex procedures where EN has most responsibility. No CoC measure	In-house GP with clinical manager. RN/EN team leader for PCAs who dispense medications instead of RN	Hospitalisation ED presentation A/H primary care visits. Polypharmacy Mortality Falls Carer satisfaction

Abbreviations; *A/H* after hours, *ADL* activities of daily living, *BBI* Bice-Boxerman Index, *CoC* continuity of care, *EN* enrolled nurse, *GP* general practitioners, *HBPC* Home Based Primary Care, *NP* nurse practitioners, *PCA* Personal Care Attendants, *RN* registered nurse, *SD* standard deviation Y = reported presence of ADL and cognitive impairment

^a^ Outcomes other than hospitalisation or ED presentation are reported in supplementary file 2 and mentioned briefly in results text

^b^ Comparison of HBPC vs alternate home care program not eligible for inclusion in this review

Studies examined continuity of care predominantly provided by primary care physicians [54–56] or GPs [53, 57]. Terminology varies between countries. In the USA and Canada, the term primary care physician refers to family doctors, internist, paediatricians, geriatricians and obstetrician-gynaecologists who all may provide primary medical care [58, 59]. In Australia, GPs refers to doctors who specialise in primary care and no broad term similar to primary care physicians is used [60]. In some other countries the GP speciality is referred to as family medicine [60]. No studies reported on the impact of continuity care with non-physician primary care professionals such pharmacists, dieticians, or physiotherapists. In two studies the continuity of a physician in combination with nurse practitioners was examined [54, 55].

Relational continuity was quantitatively measured in three studies, using the Bice-Boxerman continuity of care index in the Canadian home care study [56], the average number of primary care physicians seen in the retirement community study [54] and number of primary care physician visits to hospitalised aged care residents [57]. Two studies (one in home care and one in residential aged care) did not measure relational continuity [53, 55].

The comparison used also differed across the studies. In the Canadian home care study, high or medium continuity of relational care was compared against low continuity, as measured by the Bice-Boxerman Index [56]. In the other study of home care, outcomes before and after enrolment in the Canadian Home-Based Primary Care (HBPC) program were reported [55]. This study examined the effect of the program which delivered longitudinal primary care from physicians and nurse practitioners provided through home visits integrated with allied health support. Hospitalisation outcomes were reported for the period after enrolment in the program to the end of the HBPC program (minimum 31 days follow-up, total 82,247 person days) and compared to a period of 21 months before HBPC (excluding a 31-day period immediately prior to enrolment).

A USA study in a continuing care retirement community compared alternative approaches to delivering primary care but the proportion of people receiving different levels of care (i.e. aged care services or supported living) were not reported. In this study, two half-time physicians and two half-time nurse practitioners solely practiced at the retirement community and provided all clinical care including afterhours coverage [54]. This was compared to one full-time on-site nurse practitioner providing care with less frequent provision of physician primary care of between 1.5 and 2 days per week across three control sites [54].

The two residential care studies examined the effect of relational continuity of primary care differently [53, 57]. Continuity of primary care during hospitalisation was the focus in the cross-sectional USA study, which compared patient outcomes with higher frequency of the resident’s physician visits to those without visits during hospitalisation [57]. The Australian stepped wedge RCT aimed to recruit one on-site GP at each home to care for all residents, supported by a clinical manager [53]. A nurse in charge was appointed as a team leader for a small group of personal care attendants who were trained and given the responsibility for resident medication delivery. This was compared to the Australian standard care model, which was described as a continuity model, with medical care provided by external GPs. In the Australian standard care model, registered nurses have the responsibility of dispensing medications and management of complex procedural patient care such as wound dressings, while certificate II qualified or enrolled nurses manage other aspects of patient care.

Hospitalisation outcomes were reported for all included studies. Outcomes were reported as the incidence rate ratio (IRR) [53, 55], hazard ratio (HR) for time until first hospitalisation [56], rate of hospital admissions [54], or length of stay [53, 57]. Effects on primary care resource utilisation were reported in two studies; the study examining on-site primary care compared to the standard continuity model [53] and the study examining on-site provision of primary care in the USA continuing retirement community [54]. Mortality data were reported for three studies, with one reporting in-hospital mortality only [53, 54, 57]. Other outcomes reported in single studies were specialty care consults [54], medications per resident, polypharmacy, patient or family complaints and falls [53], functional score, and number of procedures performed during admission [57].

### Home care recipients

Two studies examined relational continuity of primary care in home care recipients [55, 56]. A large Canadian observational study (*n* = 178, 686) of home care recipients assessed with the Resident Assessment Instrument for Home Care (RAI-HC) examined hospital admission and ED presentation data. Home care recipients with the highest tertile of continuity of care with the same primary care physician over the study period had a lower risk of an ED presentation (high vs. low continuity hazard ratio, HR, 0.90, 95% confidence interval, CI, 0.89 to 0.92) or any hospital admission (high vs. low HR; 0.94; 95% CI 0.92–0.96) compared to the lowest continuity of care tertile (Table 2) [56]. Moderate continuity was also significantly associated with lower ED presentations and hospital admissions, but to a lesser extent than high continuity of care for ED presentations (ED presentations medium vs. low HR 0.96, 95%CI 0.94–0.98; hospital admission medium vs. low HR 0.96; 95%CI 0.94–0.98).

**Table 2:** Impact of continuity of primary care on hospital outcomes in aged care recipients

Author, Year	N	Outcome Measure	Continuity comparison	Hospitalisation			Emergency Department Presentations		
				**Point Estimate**	**95% CI**	***P*** **-value**	**Point Estimate**	**95% CI**	***P*** **-value**
**Home care**									
Jones, 2020 [56]	178,686	HR 1^st^ admission/visit	High vs. low	0.94	0.92–0.96	NR	0.90	0.89–0.92	NR
			Medium vs. low	0.96	0.94–0.98	NR	0.96	0.94–0.98	NR
McGregor, 2018 [55]	246	Adjusted IRR admission/visit ^a^	Pre-post HBPC	0.99	0.76–1.27	NR	0.91	0.72–1.15	NR
**Continuing Care Retirement Community**									
Bynum, 2011 [54]	2,468	IRR, all admissions ^b^	24/7 physicians & NPs on-site vs limited on-site GP	0.55^b^	NA	< 0.05	0.36^b^	NR	< 0.001^c^
		IRR, medical admissions ^b^		0.41^b^	NA	0.002^c^			
		IRR, surgical admissions ^b^		0.77^b^	NA	0.173^c^			
**Residential care**									
Haines, 2020 [53]	NR (15 sites)	IRR, unplanned – Primary ITT analysis ^d^	In house GP + changed nurse roles ^e^ vs Aust standard (“continuity model”)	0.74	0.56–0.96	0.024^f^	0.81^g^	0.66–1.01	0.06^f^
		IRR, unplanned – contamination adjusted ^d^		0.52	0.41–0.64	< 0.001	0.53	0.43–0.66	< 0.001
		Length of hospital stay- IRR, Primary ITT analysis ^d^		0.87	0.79–0.97	0.007^f^	NA	NA	NA
		Length of hospital stay- IRR, contamination adjusted ^d^		0.44	0.30–0.63	< 0.001			
Susman, 1989 [57]	335	Mean length of stay (days)	0 Physician visits	9.6 days	NR	< 0.005^h^	NA	NA	NA
			1 Physician visits	11.4 days					
			2 Physician visits	11.8 days					
			≥ 3 Physician visits	13.1 day					

*Abbreviations: CI* confidence interval, *GP* general practitioner, *HBPC* Home Based Primary Care, *HR* hazard ratio, *IRR* incidence rate ratio, *ITT* Intention-to-treat, *NA* not available (not calculable), *NPs* nurse practitioners, *NR* not reported, *NS* not significant

^a^ Adjusted for age, male, higher CHESS score, higher MAPLe score and living alone variables

^b^ Rate ratio of site D vs weighted average of control sites A-C, calculated by reviewers

^c^ P as reported by authors for comparison of rates across three control & one intervention sites

^d^ The primary analysis was ITT; the contamination adjusted ITT analysis adjusted for intervention sites according to whether a GP was employed for more than half of each nine-week block

^e^ Implementation difficulties due to GP recruitment affected four out of 15 sites

^f^ Results from pre-specified secondary analysis (with 54-week pre-trial retrospective period & 54-week post-trial follow-up in addition to 90-week trial period) were consistent

^g^ Unplanned hospital transfers

^h^ P < 0.005 for with vs without physician visits (length of stay dichotomous data 9.6 vs 12.5 days); measure of variation not reported

The provision of high continuity of care was associated with lower ED presentations for those with the lowest levels of cognitive impairment (HR 0.89, 95% CI 0.86–0.91), with a trend toward less impact for those who had moderate (HR 0.93, 95%CI 0.91–0.95) or high cognitive impairment (HR 0.93, 95%CI 0.87–0.99) (Additional file 2 Table S1). This was not observed for hospital admissions (Additional file 2 Table S1).

A small study of home care recipients (*n* = 246) who received a Home-Based Primary Care program comprising longitudinal care from physicians and nurse practitioners in Canada did not find any significant differences in hospital admission after provision of the program (Table 2) [55]. No quantitative measures of relational continuity of care were reported.

Greater relational continuity of primary care probably reduces hospital admissions and ED presentations for home care recipients (GRADE moderate certainty evidence; see Additional file 2 Table S2 for GRADE evidence ratings).

### On-site primary care teams for relational continuity in residential settings

Two studies examined on-site primary care teams in residential aged care settings. For older adults living in a continuing care retirement community, having access to 24/7 on-site physician and nurse practitioner care increased their continuity of relational care compared to those without this service [54]. These residents saw less doctors (mean 3.2 vs. 5.8 doctors) and fewer saw more than 10 doctors (5.9% vs. 15.2%). The onsite intervention was associated with fewer ED presentations (0.16 vs 0.40 presentations per person year; IRR 0.36), medical admissions (6.8 vs 16.6 admissions; IRR 0.41) and total hospitalisations (15 vs. 27 hospitalisations; IRR 0.55) compared to sites with limited on-site physician care. Surgical hospitalisations were not lower with the onsite model (8.1 vs 10.5). For hospitalised residents the onsite primary care model was associated with lower in-hospital mortality (5.1% on-site primary care compared to 14.5% limited on-site primary care). This onsite primary care model also had lower primary care visits per person per year (4.6 vs. 7.9 visits per person year), less medical specialist visits (3 vs. 7.5 visits per person year) and more visits to nurse practitioner and physician assistants (4.1 versus 2.1 visits per person year; Additional file 2 Table S1) [54].

Primary care provision through on-site GPs employed by the residential aged care facility, supported by a clinical nurse manager, was examined in an Australian stepped-wedge cluster randomised trial [53]. The control arm in this trial was described as the “continuity model”, i.e. standard care in Australia, where residents ideally continue to see their community GP. However, after admission to residential care, a resident’s continuity with their GP is likely to be low, with alternative arrangements for care being made or a change of GPs occurring [28]. No measure of relational continuity was used in this study so the degree to which residents achieved continuity with their pre-admission GP is not known.

The trial reported standard intention-to-treat (ITT) and contamination-adjusted analyses due to lack of intervention fidelity, the trialists were unable to recruit GPs to work in four of fifteen homes. GPs were present for five or more weeks in 91/148 (61%) nine-week site blocks, although the nursing care component of the intervention was implemented across all aged care facilities.

The on-site primary care model was associated with fewer unplanned hospital transfers (IRR 0.81; 95%CI 0.66, 1.01), unplanned hospital admissions (IRR 0.74; 95%CI 0.56, 0.96) and shorter hospital length of stay (IRR 0.87; 95%CI 0.79, 0.97) than the standard primary care approach for aged care residents in the primary ITT analysis (Table 2) [53]. These findings were consistent in contamination-adjusted ITT analyses which took account of the lack of fidelity of the intervention (see Table 2 and Additional file 2 Table S1). Afterhours GP call outs with the onsite model did not differ between models in the primary analysis (IRR 0.84; 95%CI 0.42,1.68; Additional file 2 Table S1). However, in the adjusted analysis the onsite model had significantly fewer out of hours call outs (IRR 0.54; 95%CI 0.36, 0.80). Those receiving onsite GP care had a non-statistically significantly higher mortality rate compared to standard control sites during the intervention period (primary analysis IRR 1.31; 95% CI 0.94–1.82) that was statistically significant in a pre-specified secondary analysis which included 54-weeks of retrospective pre-trial and post-trial follow up data, capturing mortality beyond the implementation period of the onsite GP care intervention (secondary analysis IRR 1.39, 95%CI 1.03–1.88) [53, 61].

There was a higher rate of falls in the contamination-adjusted analysis (IRR 1.37, 95% CI 1.20–1.58) with the on-site GP model, which was not statistically significant in the primary analysis (IRR 1.05, 95%CI 0.94–1.18; Additional file 2 Table S1) [53]. The number of patient or family complaints (IRR 0.87, 95%CI 0.42, 1.76) did not differ between the on-site GP and standard care model according to the ITT analyses, but were lower in the onsite model when adjusted for contamination (IRR 0.46; 95%CI 0.33, 0.63; Additional file 2 Table S1) [53]. There were no differences between the models for risk of polypharmacy and the number of medications prescribed per resident (Additional file 2 Table S1).

The benefit of on-site primary care teams provided as a relational continuity approach in residential aged care overall is considered uncertain as whilst there may be benefits in terms of lower hospitalisations and ED presentations, there may also be harms in terms of higher mortality and falls (GRADE low level evidence).

### Aged care residents in hospital

One older, observational study examined primary care physician continuity in hospitalised aged care residents (*n* = 335) in the USA, according to the number of visits the patients received. Overall, 61% of participants were visited by their primary care physician during their hospitalisation [57]. Patients who were visited by their primary care physician had a longer length of stay than those that did not receive visits (12.5 days vs 9.6 days, *P* < 0.005; Table 2) [57]. Those with physician visits also had a greater decrease in function score (-6.3 vs. -2.4, scale range 75 to -80, *p* < 0.05; Additional file 2 Table S1) [57]. The number of physician visits was not associated with mortality, mean change in functional score, number of complications or procedures at discharge (Additional file 2 Table S1). The certainty of evidence was considered very low according to GRADE criteria, thus there is uncertainty in this finding.

## Discussion

This systematic review has demonstrated that approaches impacting on the continuity of primary care can have an impact on hospitalisation rates and ED presentations in aged care recipients. Of the five included studies, two were conducted in home care settings, two in residential settings and the fifth of residential aged care residents whilst in hospital. All studies reported the impact of continuity on hospital admissions and three of these also on ED presentation outcomes. Only single studies, or two studies from different settings, reported the impact of continuity on other outcomes, including hospital length of stay (two studies); overall mortality (two studies); primary care, specialty or mid-level visits; out-of-hours GP call-outs; falls; polypharmacy; medications per resident; function; and number of procedures whilst in hospital. Thus the certainty of evidence for these outcomes was highly limited (considered low to very low certainty; see additional file 2).

### Home care recipients

The finding with the highest certainty of evidence was that greater continuity of relational care with a primary care physician probably reduces hospital admissions and ED presentations for home care recipients (GRADE moderate certainty of evidence).

This conclusion is driven by a large Canadian observational study of home care which demonstrated an association of high or medium continuity of care with a family physician with a 4–10 percent lower risk of ED presentations and a 4–6 percent lower risk of hospital admissions, after adjustment for potential confounding factors.

These findings are in accord with effects of higher continuity of care observed in older people generally (i.e., not specifically aged care recipients). In a large population-based study of more than three million older people, a 0.1 increase in a relational continuity index was associated with approximately a 2% lower rate of preventable hospitalisations in the USA [5]. Increased continuity in primary and specialist care (in an integrated healthcare delivery system with high informational continuity) has also been associated with fewer hospital admissions and ED presentations in a study of more than 12,000 older people with multimorbidity [21]. Increased continuity of specialist care in the Canadian home care cohort included in this review had findings consistent with that for primary care professionals, reporting an association with lower ED presentations and hospitalisations [56]. An older double-blind, randomised trial has demonstrated reduced hospital admissions, a shorter length of stay, higher patient satisfaction and fewer chest diagnostic tests in older men receiving more continuous care by providers at a veteran’s outpatient clinic [62].

### On-site primary care teams in residential settings

Whilst five studies were included in this review, the heterogeneity in settings and study cohorts meant that the body of evidence for relational continuity of care in residential settings was limited. In the two included studies, provision of primary care with on-site primary care physicians and/or nurse practitioners was also associated with lower hospital admissions and ED presentations. Both studies also demonstrated an association of on-site teams with fewer consults, i.e., lower primary care, specialty and mid-level visits in the continuity care retirement community and lower out-of-hours GP call outs in the Australian trial. However, in the Australian cluster randomised trial, the intervention also incorporated a change in the role of nurses and care workers [53]. Some possible negative outcomes from the intervention were also observed, including a decrease in “as required” medications and a possible higher risk of falls, mortality, and medication errors (an outcome not pre-specified as eligible for inclusion in this review) in some analyses [53]. Some of these outcomes are likely due to the more vigilant monitoring and reporting of adverse events but could be related to the change in the roles of staff other than the GPs in the facilities in the trial, including a shift in responsibility for dispensing medications from registered nurses to personal care attendants [61]. No data were reported on the quality of life of the residents. Similarly, another study of an Australian on-site GP model in residential aged care also demonstrated a decrease in ED presentations [63]. This study did not meet the criteria for this review, as the aim was to implement routine GP appointments, thus increasing the number of GP consultations and case conferences conducted. The benefit of providing on-site primary care teams in residential aged care as an alternative model of relational continuity is thus uncertain as whilst there may be benefits in terms of reduced hospitalisations and ED presentations, there may also be harms in terms of increased mortality and falls (GRADE low certainty evidence). The on-site GP model examined in the Australian trial has been mostly discontinued by the provider since this time, as the government reimbursement received for services is considered inadequate to cover the practitioners’ salaries [64].

### Aged care residents in hospital

The impact of primary care visits to aged care residents whilst in hospital is also uncertain (GRADE very low certainty evidence). Physician primary care professionals visiting aged care residents during hospital admission was associated with longer lengths of stay in one relatively small study [57]. Longer lengths of stay were also associated with decreased functional ability, ED admission, number of discharge diagnoses, complications and procedures. This study may be at risk of bias due to lack of adjustment for potential confounding factors, so it is likely that the longer length of stay is associated with increased complexity of the patients’ admission rather than the number of practitioner visits. Residents with longer lengths of stay may be likely to receive more visits due to the complexity of their admissions or increased opportunity for visits. The primary care professional may also be more aware of the circumstances the resident will face in the community and thus encourage a delay in discharge to home or ensure inappropriate early discharge does not occur. A study of more than 500,000 admissions of older patients in the USA also reported longer lengths of hospital stay in those who were cared for by their own primary care physicians, in addition to a higher likelihood of discharge to home and lower 30-day mortality [65]. A study of over 160, 000 Canadian adults demonstrated associations of in-hospital visits from the primary care professional with a lower risk of a composite outcome of readmission, ED presentation or death and increased use of home care services [4].

### Dementia and cognitive impairment

The protocol for this review indicated an intent to examine outcomes in people living with dementia as a subgroup analysis [36]. The large Canadian study of relational continuity in home care found that there was a trend for the association between continuity of care and ED presentations to be modified by cognitive impairment status, with a greater effect of continuity of care amongst those with better cognition [56]. However, no data on the role of cognitive impairment were reported in any of the other included studies. Given that care of people with dementia or cognitive impairment is a significant challenge within the provision of aged care services, studies specifically addressing this question are warranted.

### Potential impact of increasing continuity of primary care in aged care recipients

While not all home care recipients would currently be receiving low levels of continuity of care, it would seem likely that many do, and it should be feasible to increase the continuity of primary care for many. Although the potential avoidance of ED presentations and hospitalisations that may be achieved with increased relational continuity of primary care in this population may be small in percentage terms (4–10% of ED presentations and 4–6% of hospitalisations), such a reduction is considered clinically significant and could nevertheless lead to a significant financial savings to the healthcare system, particularly to government. For example, in Australia, approximately 35 percent of home care residents have an unplanned hospital admission or ED presentation over 90 days [66]. If a 6% reduction could be achieved, this would equate to an avoidance of hospitalisations for 2 percent of all older home care recipients over 90 days, or potentially 12,000 people annually [67]. The average cost of an ED presentation is approximately $AU 700 and hospitalisation for a frail, community-dwelling older person approximately $AU 23,000, thus this could lead to significant cost savings [68–70]. Reduced costs of health care associated with increased relational continuity of care has been demonstrated in a study of more than 100,000 community-dwelling older adults with dementia in the USA [71]. The findings of this review indicate that a similar result may be feasible with home care recipients.

### Policy approaches to increase relational continuity of care

Several initiatives to increase relational continuity, including a ‘named GP’ scheme, have been undertaken in recent years, with others planned [72–75]. However, the outcomes of such programs have been mixed, so the impact of these approaches is generally unclear [75]. Increasing relational continuity through policy approaches must attempt to overcome barriers including many primary care professionals working part-time, limited availability and reduced availability of practitioners in rural areas [30]. It is also possible that continuity with a preferred GP may be even more difficult during the coronavirus pandemic [76]. However, the increasing use of telehealth technologies may assist in maintaining some continuity [77]. Greater use of nurse practitioners may also assist in overcoming some of these barriers [78–80]. Approaches that have been proposed to increase continuity include nurse practitioners providing face-to-face care alongside a patient, with a GP providing consultation via telehealth, particularly in rural and remote areas with limited access to primary care [81]. In the residential aged care setting, nurse practitioners working with GPs to improve care co-ordination has led to better maintenance of resident quality of life [82]. Continuity of care provided by collaborative teams with strong relationships, rather than single providers, might have advantages in terms of maximising the use of individual practitioners’ strengths, with multiplicative rather than additive effects [83].

Policy initiatives that have been introduced with the intent of increasing relational continuity of care in the general community include patient enrolment or nomination of GP schemes [72, 73]. The evidence supporting the effectiveness of such schemes is inconsistent, however the question arises whether they may be more successful when applied to an aged care population [75]. In the UK, a large observational study of a ‘named GP scheme’ failed to demonstrate improvements in continuity of care or rates of unplanned hospitalisations [73]. However, in Germany a similar scheme that was linked to increased funding and more comprehensive care was associated with less hospitalisations [74]. In the USA, patient-centred medical homes (PCMHs) have been implemented in the community to increase continuity of primary care. PCMHs have been associated with modestly lower hospitalisation and rehospitalisation rates over 5-years follow-up and increased relational care continuity in PCHMs has been associated with lower ED presentations, hospitalisations and mortality [84–86]. This model of care has also been reported to increase receipt of preventative healthcare and the quality of care for people with diabetes and multimorbidity and depression [87, 88]. PCMHs specific for the care of older people with complex care needs (Geriatric Patient-Aligned Care Teams or GeriPACTs) have been implemented by the Veterans Health Administration, in part to reduce hospital readmission rates [89, 90]. In the USA, there has been an increase in the number of practices directly contracting patients, or “concierge medicine”, which is likely to further increase inequities [91].

In Australia, ongoing or existing initiatives include “Health Care Homes”, a voluntary patient enrolment scheme, and more recently General Practitioner Aged Care Access Incentive Payments have been provided for delivering services in residential aged care settings [92, 93]. There are also indirect incentives to encourage continuity of care through Medicare Benefits Schedule (MBS) GP Management Plans, Mental Health Care Plans and the newer telehealth MBS items [94, 95]. A voluntary GP enrolment scheme for people aged over 70 years is planned for introduction [75].

Despite the implementation of several approaches aimed at increasing continuity of primary care worldwide, data on the impact of such schemes specifically on aged care recipients are not readily available. New research on how to achieve higher relational continuity of care with primary care professionals for people receiving aged care services, in the context of the modern fractured workforce, is urgently needed. This should include research on the cost-effectiveness of providing higher rebates for GP home- and residential aged care facility visits to people receiving aged care services. Reimbursement at a level suitable to adequately fund consultation for people with complex, multimorbid conditions, encompassing both face-to-face patient and travel time and after-hours attendances for the attending physician should be determined and piloted with monitoring of the impact on relational continuity of care plus person-centred and resource use outcomes. Further studies on the effectiveness and cost-effectiveness of continuous care with consistent, collaborative, multidisciplinary primary care teams including nurse practitioners, general practitioners and allied health professionals in this population are also needed. The role of digital technologies in aiding improvements in continuity of care also warrants attention.

### Strengths and limitations of the review

This review focused specifically on aged care recipients. Whilst the evidence for the impact of increased continuity of care in older people generally may be much stronger, the body of evidence providing support for this effect specifically in aged care recipients was limited [7, 16, 20, 62]. This is somewhat unexpected, given the importance of providing high quality primary care services to this population. In particular, there was a lack of studies measuring relational continuity of care and reporting outcomes in residential aged care settings. Conducting studies in this setting can be challenging for many reasons including difficulties obtaining ethics approvals and the co-operation of the sector [35, 96]. Nevertheless, better understanding of the impact in this setting could be critical to driving important policy reforms.

No studies eligible for this review specifically examined the impact of increased relational continuity of care with other primary care professionals, such as nurse practitioners acting independently or allied health providers. Increased continuity of allied health in aged care settings also has the potential to improve outcomes. For example, the development of relationships and trust between physiotherapists and residents of care homes living with dementia are particularly important enablers of effective, individually tailored, functional exercise programs [97].

The current review has focused on relational continuity of primary care for older adult recipients of aged care services. There are many studies that examine continuity of care in the older population more broadly that were not eligible for inclusion in this review, as they were not focussed on aged care recipients [7, 15, 16, 20, 23, 62]. It is possible that the impact of continuity in the general older population differs to that in an aged care population for many reasons, including their different medical and functional profiles as well as differences in access to other services. Thus, the findings of this review may not be generalisable to the older population more broadly. However, if this review had used inclusion criteria of studies conducted in older people generally, a larger number of studies and possibly studies reporting on a broader range of outcomes would have been eligible for inclusion. It is also possible that there may have been a higher level of evidence for an impact of increased continuity of primary care on patient outcomes in that broader older population.

Whilst the review focussed on relational continuity of care, it is likely that increased relational continuity was accompanied by improved informational and management continuity in comparison to participants with lower relational continuity, and that all of these factors contribute to an effect on decreasing hospital admission and ED presentations [29]. There are many other complex interventions, such as case management, care co-ordination or reablement approaches, that are likely to increase relational continuity of care (as well as other types of continuity) as a component of the intervention, that were not examined in this review. Studies of these interventions generally aimed to increase referrals, access to or use of services, or integration between services rather than to increase continuity of care per se, did not measure continuity or were not specifically an aged care cohort and thus did not meet the inclusion criterion for this review [98–100]. Previous systematic reviews have also excluded organisational continuity, interventions about staffing numbers or required the reporting of quantitative continuity measures, and thus did not identify studies specifically addressing informational or management continuity that were eligible for inclusion [23, 24]. The included studies were also limited to high income countries, so there is a need for studies conducted in lower resource settings.

This review has included a comprehensive search strategy without language restrictions and included approaches to identify studies through means other than mainstream database searching to identify all relevant studies, however the omission of eligible studies, particularly in grey literature sources, is possible. Two of the included studies did not include a quantitative measure of continuity of care [53, 55]. This was not specified as inclusion criteria for this review, however studies that reported such measures provided information of more direct relevance to the research question.

## Conclusion

Hospitalisation outcomes in aged care recipients can be improved by interventions targeting primary care physician relational continuity across the care spectrum. Increased continuity of relational care with a primary care physician probably decreases hospitalisations and ED presentations for home care recipients. Despite the existence of many studies of the impact of continuity of care for older people in the general population, further research is needed to determine effective approaches in aged care recipients, particularly in residential settings. Policy approaches targeted to improving continuity of primary care for aged care recipients, that address workforce and other barriers, may therefore have cost offsets as well as improving outcomes for older people.

## Supplementary Information

**Additional file 1:** 

**Additional file 2: Table S1. **Impact of continuity of primarycare on additional included outcomes in aged care recipients. **TableS2.** GRADE ratings of certainty of evidence: Home Care (agreement of duplicateratings by two independent reviewers). **Table S3.** GRADE ratings of certainty of evidence: On-site primary care in residentialsettings (agreement of duplicate ratings by two independent reviewers). **Table S4.** GRADE ratings of certainty of evidence: Primary Physician Visits to residentsof aged care facilities during hospital admission (agreement of duplicateratings by two independent reviewers).  

## Data Availability

All data generated or analysed during this study are included in this published article [and its supplementary information files].

## Abbreviations

AMSTAR: A MeaSurement Tool to Assess systematic Reviews
CI: Confidence interval
ED: Emergency department
GP: General practitioner
GRADE: Grades of Recommendation, Assessment, Development, and Evaluation
HR: Hazard ratio
IRR: Incidence rate ratio
PRISMA: Preferred Reporting Items for Systematic Reviews and Meta-Analyses
RCT: Randomised controlled trial
UK: United Kingdom
USA: United States of America
