# Supplementary material for: Impact of relational continuity of primary care in aged care: a systematic review
Source: BMC Geriatr. 2022 Jul 14;22:579. doi: 10.1186/s12877-022-03131-2 (PMC9281225; doi:10.1186/s12877-022-03131-2)
Supplement: Supplementary file 1 — Additional file 1: [file 12877_2022_3131_MOESM1_ESM.docx]

**Impact of relational continuity of primary care in aged care: a systematic review**

# Supplementary Materials

## Search Strategies

Database: Ovid MEDLINE(R) and Epub Ahead of Print, In-Process & Other Non-Indexed Citations, Daily and Versions(R) <1946 to October 27, 2020>

Search Strategy:

--------------------------------------------------------------------------------

1 exp Geriatrics/

2 exp Aged/

3 aged*.ti,ab.

4 old*.ti,ab.

5 senior$.ti,ab.

6 elderly.ti,ab.

7 geriatric*.ti,ab.

8 1 or 2 or 3 or 4 or 5 or 6 or 7

9 exp Nursing Homes/

10 housing for the elderly/

11 residential facilities/

12 assisted living facilities/

13 group homes/

14 homes for the aged/

15 senior centers/

16 exp Home Nursing/

17 Adult Day Care Centers/

18 ("aged care" adj1 facilit*).ti,ab.

19 (residential adj2 care).ti,ab.

20 (home adj1 care).ti,ab.

21 "long?term care".ti,ab.

22 "LTC".ti,ab.

23 "social care".ti,ab.

24 respite.ti,ab.

25 (home* adj2 nursing).ti,ab.

26 (residential adj1 aged*).ti,ab.

27 (informal adj1 care).ti,ab.

28 "community nurs*".ti,ab.

29 Primary Health Care/

30 General Practice/

31 Community Health Services/

32 Community Health Workers/

33 Community Pharmacy Services/

34 Nurse Practitioners/

35 Nurse Clinicians/

36 Advanced Practice Nursing/

37 Occupational Therapists/

38 Nutritionists/

39 Physical Therapists/

40 Podiatry/

41 Psychology/

42 Audiologists/

43 Optometrists/

44 "General practi*".ti,ab.

45 "primary health*".ti,ab.

46 "family practice".ti,ab.

47 "family medicine".ti,ab.

48 "community pharmac*".ti,ab.

49 "allied health".ti,ab.

50 exp *dental care for aged/

51 "GP".ti,ab.

52 29 or 30 or 31 or 32 or 33 or 34 or 35 or 36 or 37 or 38 or 39 or 40 or 41 or 42 or 43 or 44 or 45 or 46 or 47 or 48 or 49 or 50 or 51

53 Continuity of patient care/

54 "service model".ti,ab,kw.

55 continu*.ti,ab,kw.

56 "model? of care".ti,ab,kw.

57 53 or 54 or 55 or 56

58 Home Care Services/

59 9 or 10 or 11 or 12 or 13 or 14 or 15 or 16 or 17 or 18 or 19 or 20 or 21 or 22 or 23 or 24 or 25 or 26 or 27 or 28 or 58

60 8 and 52 and 57 and 59

Database: APA PsycInfo <1806 to October Week 3 2020>

Search Strategy:

--------------------------------------------------------------------------------

1 exp Geriatrics/

2 exp Aged/

3 aged*.ti,ab.

4 old*.ti,ab.

5 senior$.ti,ab.

6 elderly.ti,ab.

7 geriatric*.ti,ab.

8 1 or 2 or 3 or 4 or 5 or 6 or 7

9 exp Nursing Homes/

10 group homes/

11 elder care/

12 long term care/

13 ("aged care" adj1 facilit*).ti,ab.

14 (residential adj2 care).ti,ab.

15 (home adj1 care).ti,ab.

16 "long?term care".ti,ab.

17 "LTC".ti,ab.

18 "social care".ti,ab.

19 respite.ti,ab.

20 (home* adj2 nursing).ti,ab.

21 (residential adj1 aged*).ti,ab.

22 (informal adj1 care).ti,ab.

23 "community nurs*".ti,ab.

24 "home care".ti,ab.

25 Residential Care Institutions/

26 assisted living/ or independent living programs/

27 "continu*".ti,ab,id.

28 "continuum of care"/

29 "model? of care".ti,ab,id.

30 "service model".ti,ab,id.

31 Primary Health Care/

32 General Practitioners/

33 Community Services/ or Health Care Services/

34 exp Pharmacists/

35 public health service nurses/

36 Occupational Therapists/

37 health personnel/ or allied health personnel/ or home care personnel/

38 Physical Therapists/

39 Psychology/

40 Audiology/

41 Optometrists/

42 "General practi*".ti,ab.

43 "primary health*".ti,ab.

44 "family practice".ti,ab.

45 "community pharmac*".ti,ab.

46 "allied health".ti,ab.

47 "GP".ti,ab.

48 31 or 32 or 33 or 34 or 35 or 36 or 37 or 38 or 39 or 40 or 41 or 42 or 43 or 44 or 45 or 46 or 47

49 27 or 28 or 29 or 30

50 Home care/

51 9 or 10 or 11 or 12 or 13 or 14 or 15 or 16 or 17 or 18 or 19 or 20 or 21 or 22 or 23 or 24 or 25 or 26 or 50

52 8 and 48 and 49 and 51

CINAHL

| **#** | **Query** | **Limiters/Expanders** | **Last Run Via** |
| --- | --- | --- | --- |
| S95 | S14 AND S49 AND S60 AND S94 | Search modes - Boolean/Phrase | Interface - EBSCOhost Research Databases Search Screen - Advanced Search Database - CINAHL |
| S94 | S61 OR S62 OR S63 OR S64 OR S66 OR S67 OR S68 OR S70 OR S71 OR S73 OR S74 OR S75 OR S76 OR S77 OR S78 OR S79 OR S80 OR S81 OR S82 OR S83 OR S84 OR S85 OR S86 OR S87 OR S88 OR S89 OR S90 OR S91 OR S92 OR S93 | Search modes - Boolean/Phrase | Interface - EBSCOhost Research Databases Search Screen - Advanced Search Database - CINAHL |
| S93 | AB "GP" | Search modes - Boolean/Phrase | Interface - EBSCOhost Research Databases Search Screen - Advanced Search Database - CINAHL |
| S92 | TI "GP" | Search modes - Boolean/Phrase | Interface - EBSCOhost Research Databases Search Screen - Advanced Search Database - CINAHL |
| S91 | MH dental care for aged | Search modes - Boolean/Phrase | Interface - EBSCOhost Research Databases Search Screen - Advanced Search Database - CINAHL |
| S90 | AB "allied health" | Search modes - Boolean/Phrase | Interface - EBSCOhost Research Databases Search Screen - Advanced Search Database - CINAHL |
| S89 | TI "allied health" | Search modes - Boolean/Phrase | Interface - EBSCOhost Research Databases Search Screen - Advanced Search Database - CINAHL |
| S88 | AB "community pharmac*" | Search modes - Boolean/Phrase | Interface - EBSCOhost Research Databases Search Screen - Advanced Search Database - CINAHL |
| S87 | TI "community pharmac*" | Search modes - Boolean/Phrase | Interface - EBSCOhost Research Databases Search Screen - Advanced Search Database - CINAHL |
| S86 | AB "family medicine" | Search modes - Boolean/Phrase | Interface - EBSCOhost Research Databases Search Screen - Advanced Search Database - CINAHL |
| S85 | TI "family medicine" | Search modes - Boolean/Phrase | Interface - EBSCOhost Research Databases Search Screen - Advanced Search Database - CINAHL |
| S84 | AB "family practice". | Search modes - Boolean/Phrase | Interface - EBSCOhost Research Databases Search Screen - Advanced Search Database - CINAHL |
| S83 | TI "family practice" | Search modes - Boolean/Phrase | Interface - EBSCOhost Research Databases Search Screen - Advanced Search Database - CINAHL |
| S82 | AB "primary health*" | Search modes - Boolean/Phrase | Interface - EBSCOhost Research Databases Search Screen - Advanced Search Database - CINAHL |
| S81 | TI "primary health*" | Search modes - Boolean/Phrase | Interface - EBSCOhost Research Databases Search Screen - Advanced Search Database - CINAHL |
| S80 | AB "General practi*" | Search modes - Boolean/Phrase | Interface - EBSCOhost Research Databases Search Screen - Advanced Search Database - CINAHL |
| S79 | TI "General practi*" | Search modes - Boolean/Phrase | Interface - EBSCOhost Research Databases Search Screen - Advanced Search Database - CINAHL |
| S78 | MH Optometrists | Search modes - Boolean/Phrase | Interface - EBSCOhost Research Databases Search Screen - Advanced Search Database - CINAHL |
| S77 | MH Audiologists | Search modes - Boolean/Phrase | Interface - EBSCOhost Research Databases Search Screen - Advanced Search Database - CINAHL |
| S76 | MH Psychology | Search modes - Boolean/Phrase | Interface - EBSCOhost Research Databases Search Screen - Advanced Search Database - CINAHL |
| S75 | MH Podiatry/ | Search modes - Boolean/Phrase | Interface - EBSCOhost Research Databases Search Screen - Advanced Search Database - CINAHL |
| S74 | MH Physical Therapists | Search modes - Boolean/Phrase | Interface - EBSCOhost Research Databases Search Screen - Advanced Search Database - CINAHL |
| S73 | (MH "Nutrition Services") | Search modes - Boolean/Phrase | Interface - EBSCOhost Research Databases Search Screen - Advanced Search Database - CINAHL |
| S72 | MH Nutritionists/ | Search modes - Boolean/Phrase | Interface - EBSCOhost Research Databases Search Screen - Advanced Search Database - CINAHL |
| S71 | MH Occupational Therapists/ | Search modes - Boolean/Phrase | Interface - EBSCOhost Research Databases Search Screen - Advanced Search Database - CINAHL |
| S70 | (MH "Advanced Nursing Practice") | Search modes - Boolean/Phrase | Interface - EBSCOhost Research Databases Search Screen - Advanced Search Database - CINAHL |
| S69 | MH Advanced Practice Nursing | Search modes - Boolean/Phrase | Interface - EBSCOhost Research Databases Search Screen - Advanced Search Database - CINAHL |
| S68 | (MH "Clinical Nurse Specialists") | Search modes - Boolean/Phrase | Interface - EBSCOhost Research Databases Search Screen - Advanced Search Database - CINAHL |
| S67 | MH Nurse Practitioners | Search modes - Boolean/Phrase | Interface - EBSCOhost Research Databases Search Screen - Advanced Search Database - CINAHL |
| S66 | (MH "Medication Management") | Search modes - Boolean/Phrase | Interface - EBSCOhost Research Databases Search Screen - Advanced Search Database - CINAHL |
| S65 | MH Community Pharmacy Services | Search modes - Boolean/Phrase | Interface - EBSCOhost Research Databases Search Screen - Advanced Search Database - CINAHL |
| S64 | MH Community Health Workers | Search modes - Boolean/Phrase | Interface - EBSCOhost Research Databases Search Screen - Advanced Search Database - CINAHL |
| S63 | MH Community Health Services | Search modes - Boolean/Phrase | Interface - EBSCOhost Research Databases Search Screen - Advanced Search Database - CINAHL |
| S62 | (MH "Family Practice") | Search modes - Boolean/Phrase | Interface - EBSCOhost Research Databases Search Screen - Advanced Search Database - CINAHL |
| S61 | MH Primary Health Care | Search modes - Boolean/Phrase | Interface - EBSCOhost Research Databases Search Screen - Advanced Search Database - CINAHL |
| S60 | S50 OR S51 OR S52 OR S53 OR S54 OR S55 OR S56 OR S57 OR S58 OR S59 | Search modes - Boolean/Phrase | Interface - EBSCOhost Research Databases Search Screen - Advanced Search Database - CINAHL |
| S59 | "service model" | Search modes - Boolean/Phrase | Interface - EBSCOhost Research Databases Search Screen - Advanced Search Database - CINAHL |
| S58 | AB "service model" | Search modes - Boolean/Phrase | Interface - EBSCOhost Research Databases Search Screen - Advanced Search Database - CINAHL |
| S57 | TI "service model" | Search modes - Boolean/Phrase | Interface - EBSCOhost Research Databases Search Screen - Advanced Search Database - CINAHL |
| S56 | "model* of care" | Search modes - Boolean/Phrase | Interface - EBSCOhost Research Databases Search Screen - Advanced Search Database - CINAHL |
| S55 | AB "model* of care" | Search modes - Boolean/Phrase | Interface - EBSCOhost Research Databases Search Screen - Advanced Search Database - CINAHL |
| S54 | TI 'model* of care" | Search modes - Boolean/Phrase | Interface - EBSCOhost Research Databases Search Screen - Advanced Search Database - CINAHL |
| S53 | MH Continuity of patient care/ | Search modes - Boolean/Phrase | Interface - EBSCOhost Research Databases Search Screen - Advanced Search Database - CINAHL |
| S52 | "continu*" | Search modes - Boolean/Phrase | Interface - EBSCOhost Research Databases Search Screen - Advanced Search Database - CINAHL |
| S51 | AB "continu*" | Search modes - Boolean/Phrase | Interface - EBSCOhost Research Databases Search Screen - Advanced Search Database - CINAHL |
| S50 | TI "continu*" | Search modes - Boolean/Phrase | Interface - EBSCOhost Research Databases Search Screen - Advanced Search Database - CINAHL |
| S49 | S15 OR S16 OR S19 OR S20 OR S21 OR S22 OR S24 OR S25 OR S26 OR S27 OR S28 OR S29 OR S30 OR S31 OR S32 OR S33 OR S34 OR S35 OR S36 OR S37 OR S38 OR S39 OR S40 OR S41 OR S42 OR S43 OR S44 OR S45 OR S46 OR S47 OR S48 | Search modes - Boolean/Phrase | Interface - EBSCOhost Research Databases Search Screen - Advanced Search Database - CINAHL |
| S48 | AB "home care" | Search modes - Boolean/Phrase | Interface - EBSCOhost Research Databases Search Screen - Advanced Search Database - CINAHL |
| S47 | TI "home care" | Search modes - Boolean/Phrase | Interface - EBSCOhost Research Databases Search Screen - Advanced Search Database - CINAHL |
| S46 | AB "community nurs*" | Search modes - Boolean/Phrase | Interface - EBSCOhost Research Databases Search Screen - Advanced Search Database - CINAHL |
| S45 | TI "community nurs*" | Search modes - Boolean/Phrase | Interface - EBSCOhost Research Databases Search Screen - Advanced Search Database - CINAHL |
| S44 | AB (informal N1 care) | Search modes - Boolean/Phrase | Interface - EBSCOhost Research Databases Search Screen - Advanced Search Database - CINAHL |
| S43 | TI (informal N1 care) | Search modes - Boolean/Phrase | Interface - EBSCOhost Research Databases Search Screen - Advanced Search Database - CINAHL |
| S42 | AB (residential N1 aged*) | Search modes - Boolean/Phrase | Interface - EBSCOhost Research Databases Search Screen - Advanced Search Database - CINAHL |
| S41 | TI (residential N1 aged*) | Search modes - Boolean/Phrase | Interface - EBSCOhost Research Databases Search Screen - Advanced Search Database - CINAHL |
| S40 | AB (home* N2 nursing) | Search modes - Boolean/Phrase | Interface - EBSCOhost Research Databases Search Screen - Advanced Search Database - CINAHL |
| S39 | TI (home* N2 nursing) | Search modes - Boolean/Phrase | Interface - EBSCOhost Research Databases Search Screen - Advanced Search Database - CINAHL |
| S38 | AB respite | Search modes - Boolean/Phrase | Interface - EBSCOhost Research Databases Search Screen - Advanced Search Database - CINAHL |
| S37 | TI respite | Search modes - Boolean/Phrase | Interface - EBSCOhost Research Databases Search Screen - Advanced Search Database - CINAHL |
| S36 | AB "social care" | Search modes - Boolean/Phrase | Interface - EBSCOhost Research Databases Search Screen - Advanced Search Database - CINAHL |
| S35 | TI "social care" | Search modes - Boolean/Phrase | Interface - EBSCOhost Research Databases Search Screen - Advanced Search Database - CINAHL |
| S34 | AB "LTC" | Search modes - Boolean/Phrase | Interface - EBSCOhost Research Databases Search Screen - Advanced Search Database - CINAHL |
| S33 | TI "LTC" | Search modes - Boolean/Phrase | Interface - EBSCOhost Research Databases Search Screen - Advanced Search Database - CINAHL |
| S32 | AB "long*term care" | Search modes - Boolean/Phrase | Interface - EBSCOhost Research Databases Search Screen - Advanced Search Database - CINAHL |
| S31 | TI "long*term care" | Search modes - Boolean/Phrase | Interface - EBSCOhost Research Databases Search Screen - Advanced Search Database - CINAHL |
| S30 | AB (home N1 care) | Search modes - Boolean/Phrase | Interface - EBSCOhost Research Databases Search Screen - Advanced Search Database - CINAHL |
| S29 | TI (home N1 care) | Search modes - Boolean/Phrase | Interface - EBSCOhost Research Databases Search Screen - Advanced Search Database - CINAHL |
| S28 | AB (residential N2 care) | Search modes - Boolean/Phrase | Interface - EBSCOhost Research Databases Search Screen - Advanced Search Database - CINAHL |
| S27 | TI (residential N2 care) | Search modes - Boolean/Phrase | Interface - EBSCOhost Research Databases Search Screen - Advanced Search Database - CINAHL |
| S26 | AB ("aged care" N1 facilit*) | Search modes - Boolean/Phrase | Interface - EBSCOhost Research Databases Search Screen - Advanced Search Database - CINAHL |
| S25 | TI ("aged care" N1 facilit*) | Search modes - Boolean/Phrase | Interface - EBSCOhost Research Databases Search Screen - Advanced Search Database - CINAHL |
| S24 | (MH "Adult Day Center (Saba CCC)") OR (MH "Hospice (Saba CCC)") | Search modes - Boolean/Phrase | Interface - EBSCOhost Research Databases Search Screen - Advanced Search Database - CINAHL |
| S23 | MH Adult Day Care Centers/ | Search modes - Boolean/Phrase | Interface - EBSCOhost Research Databases Search Screen - Advanced Search Database - CINAHL |
| S22 | MH Home Nursing | Search modes - Boolean/Phrase | Interface - EBSCOhost Research Databases Search Screen - Advanced Search Database - CINAHL |
| S21 | (MH "Senior Centers") | Search modes - Boolean/Phrase | Interface - EBSCOhost Research Databases Search Screen - Advanced Search Database - CINAHL |
| S20 | MW homes for the aged/ | Search modes - Boolean/Phrase | Interface - EBSCOhost Research Databases Search Screen - Advanced Search Database - CINAHL |
| S19 | (MH "Assisted Living") OR (MH "Housing for the Elderly") | Search modes - Boolean/Phrase | Interface - EBSCOhost Research Databases Search Screen - Advanced Search Database - CINAHL |
| S18 | MH group homes/ | Search modes - Boolean/Phrase | Interface - EBSCOhost Research Databases Search Screen - Advanced Search Database - CINAHL |
| S17 | MH assisted living facilities/ | Search modes - Boolean/Phrase | Interface - EBSCOhost Research Databases Search Screen - Advanced Search Database - CINAHL |
| S16 | MH residential facilities/ | Search modes - Boolean/Phrase | Interface - EBSCOhost Research Databases Search Screen - Advanced Search Database - CINAHL |
| S15 | MH Nursing Homes/ | Expanders - Apply equivalent subjects Search modes - Boolean/Phrase | Interface - EBSCOhost Research Databases Search Screen - Advanced Search Database - CINAHL |
| S14 | S1 OR S2 OR S3 OR S4 OR S5 OR S6 OR S7 OR S8 OR S9 OR S10 OR S11 OR S12 OR S13 | Search modes - Boolean/Phrase | Interface - EBSCOhost Research Databases Search Screen - Advanced Search Database - CINAHL |
| S13 | MH Geriatrics | Expanders - Apply equivalent subjects Search modes - Boolean/Phrase | Interface - EBSCOhost Research Databases Search Screen - Advanced Search Database - CINAHL |
| S12 | MH Aged | Search modes - Boolean/Phrase | Interface - EBSCOhost Research Databases Search Screen - Advanced Search Database - CINAHL |
| S11 | TI Aged | Search modes - Boolean/Phrase | Interface - EBSCOhost Research Databases Search Screen - Advanced Search Database - CINAHL |
| S10 | AB geriatric* | Search modes - Boolean/Phrase | Interface - EBSCOhost Research Databases Search Screen - Advanced Search Database - CINAHL |
| S9 | TI geriatric* | Search modes - Boolean/Phrase | Interface - EBSCOhost Research Databases Search Screen - Advanced Search Database - CINAHL |
| S8 | AB senior$ | Search modes - Boolean/Phrase | Interface - EBSCOhost Research Databases Search Screen - Advanced Search Database - CINAHL |
| S7 | TI senior$ | Search modes - Boolean/Phrase | Interface - EBSCOhost Research Databases Search Screen - Advanced Search Database - CINAHL |
| S6 | AB old* | Search modes - Boolean/Phrase | Interface - EBSCOhost Research Databases Search Screen - Advanced Search Database - CINAHL |
| S5 | TI old* | Search modes - Boolean/Phrase | Interface - EBSCOhost Research Databases Search Screen - Advanced Search Database - CINAHL |
| S4 | AB aged* | Search modes - Boolean/Phrase | Interface - EBSCOhost Research Databases Search Screen - Advanced Search Database - CINAHL |
| S3 | TI aged* | Search modes - Boolean/Phrase | Interface - EBSCOhost Research Databases Search Screen - Advanced Search Database - CINAHL |
| S2 | AB elderly | Search modes - Boolean/Phrase | Interface - EBSCOhost Research Databases Search Screen - Advanced Search Database - CINAHL |
| S1 | TI elderly | Search modes - Boolean/Phrase | Interface - EBSCOhost Research Databases Search Screen - Advanced Search Database - CINAHL |

**HTA Database**

("Geriatrics"[mh] OR "Aged"[mh] OR elderly[title, abs]) AND ("Residential Facilities"[mhe] OR "Nursing Homes"[mhe] OR "Home Care Services"[mh] OR "Long-Term Care"[mh] OR "Respite Care"[mh] OR residential care[title,abs] OR nursing home[title,abs] OR home care[title,abs] OR long term care title,abs]) AND ("Continuity of Patient Care"[mh] OR continu* [title,abs,keywords] OR model of care [title,abs,keywords]) AND ("Primary Health Care"[mh] OR “"Health Services for the Aged"[mh] OR “Community Health Services”[mh] OR “Dental Care for Aged”[mh] OR "General Practice"[mhe] OR "Allied Health Occupations"[mhe] OR "Dietetics"[mh] OR "Community Pharmacy Services"[mh] OR "Community Health Nursing"[mh] OR allied health[title,abs] OR general practice[title,abs] OR family practice[title,abs] OR “GP” [title,abs] OR community pharmacy[title,abs])

**Cochrane SR and Trials**

ID Search

#1 MeSH descriptor: [Geriatrics] this term only

#2 MeSH descriptor: [Aged] explode all trees

#3 #1 OR #2

#4 #3 OR old* OR senior* OR elderly:ti,ab

#5 MeSH descriptor: [Nursing Homes] explode all trees

#6 care NEXT facilit*:ti,ab

#7 (residential NEAR/2 care):ti,ab

#8 (home NEAR/1 care):ti,ab

#9 long?termcare:ti,ab

#10 "LTC":ti,ab

#11 "social care":ti,ab

#12 respite:ti,ab

#13 (home* NEAR/2 nursing):ti,ab

#14 (residential NEAR/1 aged*):ti,ab

#15 (informal NEAR/1 care):ti,ab

#16 community NEXT nurs*:ti,ab

#17 #5 OR #6 OR #7 OR #8 OR #9 OR #10 OR #11 OR #12 OR #13 OR #14 OR #15 OR #16

#18 continu*:ti,ab,kw

#19 MeSH descriptor: [Continuity of Patient Care] 1 tree(s) exploded

#20 (model* NEXT "of care"):ti,ab,kw

#21 "service model":ti,ab,kw

#22 #18 OR #19 OR #20 OR #21

#23 MeSH descriptor: [Primary Health Care] this term only

#24 MeSH descriptor: [General Practice] this term only

#25 MeSH descriptor: [Community Health Services] this term only

#26 MeSH descriptor: [Community Health Workers] 1 tree(s) exploded

#27 "primary health*".ti,ab

#28 "family practice".ti,ab.

#29 "community pharmac*".ti,ab.

#30 "allied health".ti,ab.

#31 "GP".ti,ab.

#32 #23 OR #24 OR #25 OR #26 OR #27 OR #28 OR #29 OR #30 OR #31

#33 #4 AND #17 AND #22 AND #32

**Trial Registries**

**ICTRP version 3.6**

- Term: primary health AND continuity care
- Term: continuity of care AND aged
- Term: general practice AND nursing home
- Term: allied health AND residential care
- Term: allied health AND nursing home
- Term: allied health AND aged care
- Term: general practice AND aged care
- Term: general practice AND residential aged care
- Term: primary care AND nursing home
- Term: family practice AND nursing home
- Term: primary care AND residential care
- Term: family practice AND residential care

**ANZCTR Trial Registration date 29 Oct 2019 to 29 Oct 2020**

- Term: continuity of care AND aged
- Term: primary health AND continuity care
- Term: general practice AND nursing home
- Term: allied health AND residential care
- Term: allied health AND nursing home
- Term: allied health AND aged care
- Term: general practice AND aged care
- Term: general practice AND residential aged care
- Term: primary care AND nursing home
- Term: family practice AND nursing home
- Term: primary care AND residential care
- Term: family practice AND residential care

**Clinical Trials.gov**

- Term: continuity of care AND aged
- Term: primary health AND continuity care
- Term: general practice AND nursing home
- Term: allied health AND residential care
- Term: allied health AND nursing home
- Term: allied health AND aged care
- Term: general practice AND aged care (Terms and synonyms automatically searched)
- Term: general practice AND residential aged care
- Term: primary care AND nursing home (Terms and synonyms automatically searched)
- Term: family practice AND nursing home (Terms and synonyms automatically searched)
- Term: primary care AND residential care (also automatically searched for principal, assisted living and main)
- Term: family practice AND residential care (also automatically search for experience)

**Open Grey**

(continuity of care OR general practice OR allied health OR primary care) AND ('older people' OR 'nursing home' OR 'residential care')

**ProQuest**

ti('continuity of care' OR 'general practice' OR 'allied health') AND ti,ab('nursing home' OR 'residential care') AND ti,ab(elderly OR old* OR geriatric)

**Google Scholar**("continuity of care") AND ("general practice" OR "allied health") AND (elderly OR geriatric OR senior* OR elderly)

## Potentially relevant studies and reasons for exclusion

| **Reason for Exclusion** | **Study specific exclusion characteristic** | **Reference** |
| --- | --- | --- |
| Wrong setting | Not primary care | Nicholson C, Shrapnel S, Dent E. How an Emergency Department based Aged Care Service supports care across the continuum. International Journal of Integrated Care (IJIC). 2018;18:1-2.  Shanahan SK, Fergus L. engAGE - Improving Outcomes for Older People in Hawkes Bay, New Zealand. International Journal of Integrated Care (IJIC). 2018;18:1-2. |
| Wrong population | Participants not receiving aged care services (or no information on receipt of aged care services reported). | Knight, J. C., et al. (2009). "Does higher continuity of family physician care reduce hospitalizations in elderly people with diabetes?" Population health management 12(2): 81-86.  Barker, I., et al. (2017). "Association between continuity of care in general practice and hospital admissions for ambulatory care sensitive conditions: cross sectional study of routinely collected, person level data." BMJ 356.  Bayliss, E. A., et al. (2015). "Effect of continuity of care on hospital utilization for seniors with multiple medical conditions in an integrated health care system." Annals of family medicine. 13(2): 123-129.  Beales, J. L. and T. Edes (2009). "Veteran's Affairs Home Based Primary Care." Clinics in geriatric medicine 25(1): 149-154.  Brener, S. S., et al. (2016). "Association between in-hospital supportive visits by primary care physicians and patient outcomes: A population-based cohort study." Journal of Hospital Medicine 11(6): 418-424.  Cheng, S.-H. and C.-C. Chen (2014). "Effects of Continuity of Care on Medication Duplication Among the Elderly." Medical Care 52(2).  Keating, P., et al. (2008). "Reducing unplanned hospital admissions and hospital bed days in the over 65 age group: results from a pilot study." Journal of Integrated Care 16(1): 3-8.  Lei, L. (2020). Continuity of Care and Health Care Utilization and Cost Among Community-dwelling Older Veterans with Dementia. Ann Arbor, University of Rochester: 198.  Worrall G, Knight J. Continuity of care is good for elderly people with diabetes: retrospective cohort study of mortality and hospitalization. Canadian Family Physician. 2011;57(1):e16-e20.  Rosenblatt, R. A., et al. (2000). "The effect of the doctor-patient relationship on emergency department use among the elderly." Am J Public Health 90(1): 97-102.  Pandhi N, DeVoe JE, Schumacher JR, Bartels C, Thorpe CT, Thorpe JM, et al. Preventive service gains from first contact access in the primary care home. Journal of the American Board of Family Medicine : JABFM. 2011;24(4):351-9.  Salisbury, C., et al. (2019). "A patient-centred intervention to improve the management of multimorbidity in general practice: the 3D RCT." Health Service and Delivery Research.  Wasson, J. H., et al. (1984). "Continuity of outpatient medical care in elderly men. A randomized trial." Jama 252(17): 2413-2417.  Weiss, L. J. and J. Blustein (1996). "Faithful patients: the effect of long-term physician-patient relationships on the costs and use of health care by older Americans." American journal of public health 86(12): 1742-1747.  White, L. (2017). "Continuity of care in older adults with multiple chronic conditions." Dissertation Abstracts International Section A: Humanities and Social Sciences 78 |
| Wrong intervention | Comparing two different care provision models with no stated intent to examine continuity of care | Pain, T., et al. (2014). "AgedCare+GP: description and evaluation of an in-house model of general practice in a residential aged-care facility." Australian journal of primary health 20(3): 224-227.  Morales-Asencio JM, Gonzalo-Jiménez E, Martin-Santos FJ, Morilla-Herrera JC, Celdráan-Mañas M, Carrasco AM, et al. Effectiveness of a nurse-led case management home care model in Primary Health Care. A quasi-experimental, controlled, multi-centre study. BMC health services research. 2008;8:193.  Chappell HW, Murrell D. Nursing home patients: liaison nurse visits influence recidivism. Journal of gerontological nursing. 1994;20(5):33-48.  Applebaum R, Straker J, Mehdizadeh S, Warshaw G, Gothelf E. Using high-intensity care management to integrate acute and long-term care services: substitute for large scale system reform? Care management journals : Journal of case management ; The journal of long term home health care. 2002;3(3):113-9. |
| Wrong comparator | No comparator | Lei, L. (2020). Continuity of Care and Health Care Utilization and Cost Among Community-dwelling Older Veterans with Dementia. Ann Arbor, University of Rochester: 198.  Rothera I, Jones R, Harwood R, Avery A, Waite J. General practitioner contacts with older residents in nursing and residential homes. The European journal of general practice. 2003;9(4):141-2. |
| Wrong outcome | Only qualitative outcomes reported | Gallagher, N., et al. (2013). "Service users’ and caregivers’ perspectives on continuity of care in out-of-hours primary care." Qualitative Health Research 23(3): 407-421. |

## List of Appraisal Criteria based on each study design

| **The Cochrane Collaboration Risk of Bia 2.0 tool for randomised controlled trials** |
| --- |
| **Risk of bias arising from the randomisation process** |
| Was the allocation sequence random? |
| Was the allocation sequence concealed until participants were enrolled and assigned to interventions? |
| Did baseline differences between intervention groups suggest a problem with the randomisation process? |
| Risk of bias judgement (Low/ High/ Some concerns) |
| What is the predicted direction of bias arising from the randomisation process? |
| **Risk of bias due to deviations from the intended interventions (effect of assignment to intervention)** |
| 2.1 Were participants aware of their assigned intervention during the trial? |
| 2.2 Were carers and people delivering the interventions aware of participants' assigned intervention during the trial? |
| 2.3 If Y/PY/NI to 2.1 or 2.2: Were there deviations from the intended intervention that arose because of the trial context? |
| 2.4 If Y/PY to 2.3: Were these deviations likely to have affected the outcome? |
| 2.5 If Y/PY/NI to 2.4: Were these deviations from intended intervention balanced between groups? |
| 2.6 Was an appropriate analysis used to estimate the effect of assignment to intervention? |
| 2.7 If N/PN/NI to 2.6: Was there potential for a substantial impact (on the result) of the failure to analyse participants in the group to which they were randomized? |
| Risk of bias judgement (Low/ High/ Some concerns) |
| What is the predicted direction of bias to deviations from intended interventions? |
| **Risk of bias due to deviations from the intended interventions (effect of adhering to intervention)** |
| Were participants aware of their assigned intervention during the trial? |
| Were carers and people delivering the interventions aware of participants assigned intervention during the trial? |
| IF Y/PY/NI to 2.1 to 2.2. Were important non-protocol interventions balanced across intervention groups? |
| (if applicable): Were there failures in implementing the intervention that could have affected the outcome? |
| (if applicable): Was there non-adherence to the assigned intervention regimen that could have affected participants' outcomes? |
| If N/PN/NI to 2.3, or Y/PY/NI to 2.4 or 2.5: Was an appropriate analysis used to estimate the effect of adhering to intervention? |
| Risk of bias judgement (Low/ High/ Some concerns) |
| What is the predicted direction of bias due to deviations from intended interventions? |
| **Risk of bias due to missing outcome data** |
| 3.1 Were data for this outcome available for all, or nearly all, participants randomised? |
| 3.2 If N/PN/NI to 3.1: Is there evidence that the result was not biased by missing outcome data? |
| 3.3 If N/PN to 3.2: could missingness in the outcome depend on its true value? |
| 3.4 If Y/PY/NI to 3.3: Is it likely that missingness in the outcome depended on its true value? |
| Risk of bias judgement (Low/ High/ Some concerns) |
| What is the predicted direction of bias due to missing outcome data? |
| **Risk of bias in measurement of the outcome** |
| 4.1 Was the method of measuring the outcome inappropriate? |
| 4.2 Could measurement or ascertainment of the outcome have differed between intervention groups? |
| 4.3 If N/PN/NI to 4.1 and 4.2: Were outcome assessors aware of the intervention received by study participants? |
| 4.4 If Y/PY/NI to 4.3: Could assessment of the outcome have been influenced by knowledge of intervention received? |
| 4.5 If Y/PY/NI to 4.4: Is it likely that assessment of the outcome was influenced by knowledge of intervention received? |
| Risk of bias judgement (Low/ High/ Some concerns) |
| What is the predicted direction of bias in measurement of the outcome? |
| **Risk of bias in selection of the reported result** |
| 5.1 Were the data that produced this result analysed in accordance with a pre-specified analysis plan that was finalised before unblinded outcome data were available for analysis? |
| 5.2 Is the numerical result being assessed likely to have been selected, on the basis of the results, from multiple eligible outcome measurements within the outcome domain? |
| 5.3 Is the numerical result being assessed likely to have been selected, on the basis of the results, from multiple eligible analyses of the data? |
| Risk of bias judgement (Low/ High/ Some concerns) |
| What is the predicted direction of bias due to selection of the reported result? |

| **Joanna Briggs Institute Risk of Bias Tool for Cohort Studies** |
| --- |
| 1. Were the two groups similar and recruited from the same population? |
| 2. Were the exposures measured similarly to assign people to both exposed and unexposed groups? |
| 3. Was the exposure measured in a valid and reliable way? |
| 4. Were confounding factors identified? |
| 5. Were strategies to deal with confounding factors stated? |
| 6. Were the groups/participants free of the outcome at the start of the study (or at the moment of exposure)? |
| 7. Were the outcomes measured in a valid and reliable way? |
| 8. Was the follow up time reported and sufficient to be long enough for outcomes to occur? |
| 9. Was follow up complete, and if not, were the reasons to loss to follow up described and explored? |
| 10. Were strategies to address incomplete follow up utilised? |
| 11. Was appropriate statistical analysis used? |

| **Joanna Briggs Institute Risk of Bias Tool for Cross-sectional Studies** |
| --- |
| 1. Were the criteria for inclusion in the sample clearly defined? |
| 2. Were the study subjects and the setting described in detail? |
| 3. Was the exposure measured in a valid and reliable way? |
| 4. Were objective, standard criteria used for measurement of the condition? |
| 5. Were confounding factors identified? |
| 6. Were strategies to deal with confounding factors stated? |
| 7. Were the outcomes measured in a valid and reliable way? |
| 8. Was appropriate statistical analysis used? |
