# Supplementary material for: Impact of relational continuity of primary care in aged care: a systematic review
Source: BMC Geriatr. 2022 Jul 14;22:579. doi: 10.1186/s12877-022-03131-2 (PMC9281225; doi:10.1186/s12877-022-03131-2)
Supplement: Supplementary file 2 — Additional file 2: Table S1. Impact of continuity of primarycare on additional included outcomes in aged care recipients. TableS2. GRADE ratings of certainty of evidence: Home Care (agreement of duplicateratings by two independent reviewers). Table S3. GRADE ratings of certainty of evidence: On-site primary care in residentialsettings (agreement of duplicate ratings by two independent reviewers). Table S4. GRADE ratings of certainty of evidence: Primary Physician Visits to residentsof aged care facilities during hospital admission (agreement of duplicateratings by two independent reviewers). [file 12877_2022_3131_MOESM2_ESM.docx]

**Impact of relational continuity of primary care in aged care: a systematic review**

Table S1. Impact of continuity of primary care on additional included outcomes in aged care recipients

| **Author, Year** | **Participants** | | **Comparison** | **Outcome** | **Measure** | **Value (Point Estimate)** | **95% CI** | **P-value** |
| --- | --- | --- | --- | --- | --- | --- | --- | --- |
| **Home Care** | |  | |  |  |  |  |  |

| Jones, 2020 (1) | 178,688 | High vs. low continuity on risk of hospitalisation | Cognition effect | CPS 0-1 (least impaired)  CPS 2-3  CPS 4-6 (most impaired) | 0.92  0.97  0.97 | 0.89-0.95  0.94-1.00  0.89-1.06 | 0.11 ^a^ |
| --- | --- | --- | --- | --- | --- | --- | --- |
|  |  | High vs. low continuity on risk of emergency department presentations | Cognition effect | CPS 0-1 (least impaired)  CPS 2-3  CPS 4-6 (most impaired) | 0.89  0.93  0.93 | 0.86-0.91  0.91-0.95  0.87-0.99 | 0.031^a^ |
| McGregor, 2018(2) | 246 | Pre- post- HBPC | Days in hospital | Difference, rate per 1000 person days, unadjusted | -2.3 |  | 0.719 |

| **Continuing Care Retirement Community** | | |  |  |  |  |  |
| --- | --- | --- | --- | --- | --- | --- | --- |
| Bynum, 2011(3) | 2,468 | 24/7 physicians & NPs on-site  vs limited on-site GP | Primary care visits  Specialty care visits  Mid-level visits | Visits per person year | 4.6 vs 7.9  3 vs.7.5  4.1 vs. 2.1 | NR  NR  NR | <0.001  <0.001  <0.001 |
|  |  |  | Mortality in hospital | Percent of death occurring in hospital ^b^ | 5.1 vs. 14.5 | NR | 0.004 |

| **Author, Year** | **Participants** | **Comparison** | **Outcome** | **Measure** | **Value (Point Estimate)** | | **95% CI** | | **P-value** | |  |
| --- | --- | --- | --- | --- | --- | --- | --- | --- | --- | --- | --- |
| **Residential care** | | |  |  | | | |  | |  | |
| Haines, 2020 (4) | Unclear  (15 sites) | In house GP + changed nurse roles vs Aust standard (“continuity model”) | Out of hours GP call outs | IRR – primary ITT  IRR- contamination adjusted ITT ^c^ | | 0.84  0.54 | | 0.42, 1.68  0.36, 0.80 | | 0.61  0.002 | |
|  |  |  | Mortality | IRR- primary ITT  IRR- secondary ^d^ | | 1.31  1.39 | | 0.94,1.82  1.03,1.88 | | 0.12  0.030 | |
|  |  |  | Falls | IRR – primary ITT  IRR- contaminated adjusted ITT ^c^ | | 1.05  1.37 | | 0.94,1.18  1.20,1.58 | | 0.35  <0.001 | |
|  |  |  | Polypharmacy | ARR- primary ITT  ARR- secondary ^d^ | | 0  -1 | | -2, 2  -3, 1 | | 0.89  0.36 | |
|  |  |  | Medications per resident ^e^ | ARR primary ITT  ARR – secondary ^d^ | | -0.09  -0.12 | | -0.29, 0.11  -0.28, 0.05 | | 0.37  0.16 | |
|  |  |  | Change in patient/ family complaints | IRR- primary ITT  IRR- contamination adjusted ITT ^c^ | | 0.87  0.46 | | 0.42, 1.76  0.33, 0.63 | | .69  <0.001 | |
| **Physicians Visits to Care residents whilst in hospital** | | |  |  | | | |  | |  | |
| Susman, 1989 (5) | 335 | Number of Primary Physician Visits during hospital admission | Mortality (%)  Functional score (mean change) ^f^  Number of procedures  (mean) | 0 visits  1 visit  2 visits  ≥3 visits  0 visits  1 visit  ≥1visits  2 visits  ≥3 visits  0 visits  1 visit  2 visits  ≥3 visits | | 5.6  11.5  4.8  9.8  -2.4  -5.1  -6.3  -4.0  -7.3  1.6  2.2  1.6  2.1 | | NR  NR  NR  NR  NR  NR  NR  NR  NR  NR  NR  NR  NR | | NS  NS  NS  NS  NS  NS  p<0.05  NS  NS  NS  NS  NS  NS | |

*Abbreviations*: 24/7, 24 hours, 7 days a week; ARR, absolute risk reduction; CI, confidence interval; CPS, Cognitive Performance Scale; GP, general practitioner; HBPC, home-based primary care; HR, hazard ratio; GP, general practitioner; IRR, incidence risk ratio; OBD, occupied bed days; NP, nurse practitioner; NR, not reported, NS, not significant (p>0.05; P value not reported.

^a^ P for interaction of cognitive impairment with association between continuity and hospitalisation/ED outcome. Cognitive impairment: CPS 0-1 least impaired vs CPS 4-6 most impaired

^b^ Not total mortality, thus these data were not considered with the GRADE ratings in Table S3.

^c^ Contamination-adjusted analysis considered the effect of the intervention (i.e., employing GPs at the facilities) on the intervention where the effect of an inability to employ GPs at some sites was accounted for.

^d^ Secondary analysis includes pre-specified 54-week retrospective pre-trial and 54-week post-trial follow up data.

^e^ Medications were also reported within categories of antipsychotics, as required medications and antibiotics prescribed

^f^ Rating scale based on Keene and Anderson and Gauer and Birnbom. Scores range from 75 (normal, intact function) to -80 (total functional impairment.

**References:**

1. Jones A, Bronskill SE, Seow H, Junek M, Feeny D, Costa AP. Associations between continuity of primary and specialty physician care and use of hospital-based care among community-dwelling older adults with complex care needs. PloS one. 2020;15(6):e0234205.

2. McGregor MJ, Cox MB, Slater JM, Poss J, McGrail KM, Ronald LA, et al. A before-after study of hospital use in two frail populations receiving different home-based services over the same time in Vancouver, Canada. BMC health services research. 2018;18(1):248.

3. Bynum JPW, Andrews A, Sharp S, McCollough D, Wennberg JE. Fewer hospitalizations result when primary care is highly integrated into a continuing care retirement community. Health affairs (Project Hope). 2011;30(5):975-84.

4. Haines TP, Palmer AJ, Tierney P, Si L, Robinson AL. A new model of care and in-house general practitioners for residential aged care facilities: a stepped wedge, cluster randomised trial. Medical journal of Australia. 2020;212(9):409‐15.

5. Susman J, Zervanos NJ, Byerly B. Continuity of care and outcome in nursing home patients transferred to a community hospital. Family medicine. 1989;21(2):118-21.

Table S2 GRADE ratings of certainty of evidence: Home Care (agreement of duplicate ratings by two independent reviewers)

| **Question: Does increased relational continuity of primary care improve outcomes and decrease resource use for older home care recipients?** | | | | | | | |
| --- | --- | --- | --- | --- | --- | --- | --- |
| **Quality assessment**  **(Observational evidence, commenced as low quality)** | | | | | | | **Summary of Findings** |
| **Studies**  **Participants (Follow up)** | **Risk of bias** | **Inconsistency** | **Indirectness** | **Imprecision** | **Other** | **Overall quality of evidence** | **Relative effect** (95% CI) |
|  |  |  |  |  |  |  |  |
| **Hospital admissions** | | | | | | | |
| 2 observational studies (evidence commences as low quality)  A) Jones (2020) 178, 686 (6 months)  B) McGregor (2018) 246 (82, 247 person-days) | No serious concerns ^a^ | No serious concerns | No serious concerns ^b^ | No serious concerns | Dose response gradient observed (+1) | MODERATE  ⊕⊕⊕⊖ | A) High vs Low: HR 0.94 (0.92, 0.96)  A) Medium vs Low: HR 0.96 (0.94, 0.98)  B) IRR 0.99 (0.76, 1.27) |
| **ED presentations** | | | | | | | |
| 2 observational studies  A) Jones (2020) 178, 686 (6months)  B) McGregor (2018), 246 (82,247 person days) | No serious risk of bias. | No serious concerns | No serious concerns | No serious concerns | Dose response gradient observed (+1) | MODERATE  ⊕⊕⊕⊖ | A) High vs Low: HR 0.90 (0.89, 0.92) A) Medium vs Low: HR 0.96 (0.94, 0.98)  B) IRR 0.91 (0.72, 1.15) |

Abbreviations: HR, hazard ratio; IRR, incidence rate ratio.

^a^ Jones et al (2020) is at low risk of selection, measurement, confounding, contamination, and reporting bias. Follow-up not clearly reported, although unlikely to be significant attrition as data is from national health administrative databases. Whilst there are some concerns with McGregor, this study provides only a minor contribution to the evidence.

^b^ Whilst the main study providing evidence is Canadian, this study has a similar healthcare system to many countries with universal health and social care. McGregor provides only a minor contribution to the evidence

Table S3 GRADE ratings of certainty of evidence: On-site primary care in residential settings (agreement of duplicate ratings by two independent reviewers)

| **Question: Does increased relational continuity of primary care through on-site primary care in residential settings improve outcomes and decrease resource use for older aged care recipients?**  Conclusion: The benefit of providing on-site primary care teams in residential aged care is uncertain as whilst there may be benefits in terms of reduced hospitalisations and ED visits, there may also be harms in terms of increased mortality and falls (Low certainty evidence) | | | | | | | |
| --- | --- | --- | --- | --- | --- | --- | --- |
| **Quality assessment**  **(RCT evidence, commenced as high quality)** | | | | | | | **Summary of Findings** |
| **Studies**  **Participants** | **Risk of bias** | **Inconsistency** | **Indirectness** | **Imprecision** | **Other** | **Overall quality of evidence** | **Relative effect** (95% CI) |
|  |  |  |  |  |  |  |  |
| **Hospital admissions** | | | | | | | |
| 1 RCT (Haines, 2020) 15 sites, 6610 OBDs  1 observational study (Bynum, 2011) N=2, 468 | Serious concerns (-1) ^a^ | No serious concerns | No serious concerns | No serious concerns | No serious concerns | MODERATE  ⊕⊕⊕⊖ | Unplanned admissions RCT 0.74 (0.56 – 0.96) (all 3 analyses significant reduction)  Bynum IRR 0.55 (15 vs 27 P<0.05) |
| **ED presentations** | | | | | | | |
| 1 RCT (Haines, 2020) 15 sites, 6610 OBDs  1 observational study (Bynum, 2011) N=2, 468 | Serious concerns (-1) ^a^ | No serious concerns | No serious concerns | Some imprecision exists (-1) ^b^ | No serious concerns | LOW  ⊕⊕⊖⊖ | ITT RCT 0.81 (0.66, 1.01), P = 0.06; secondary 0.87 (0.72–1.04); contamination-adjusted ITT 0.53 (0.43-0.66)  Bynum IRR 0.36 (0.16 vs 0.40 P<0.001) |
| **Mortality** | | | | | | | |
| 1 RCT (Haines, 2020), 15 sites, 6610 OBDs | Serious concerns (-1) ^a^ | No serious concerns ^c^ | No serious concerns | Serious concerns (-1) ^d^ | No serious concerns | LOW  ⊕⊕⊖⊖ | Primary ITT IRR 1.31 (0.94, 1.82, P = 0.12)  Secondary analysis IRR 1.39 (1.03, 1.88, P = 0.03)  (no contamination-adjusted analysis) |
| **Falls** | | | | | | | |
| 1 RCT (Haines, 2020), 15 sites, 6610 OBDs | Serious concerns (-1) ^a^ | No serious concerns ^e^ | No serious concerns | No serious concerns | No serious concerns | LOW  ⊕⊕⊖⊖ | Contamination-adjusted ITT IRR 1.37 (1.20, 1.58, P = <0.001)  Primary ITT 1.05 (0.94-1.18, P = 0.35) & secondary analysis 1.03 (0.94, 1.14, P =0.5) not significant  (Authors conclude increased falls) |

Abbreviations: IRR, incidence rate ratio; ITT, intention-to-treat; RCT, randomised controlled trial.

^a^ Unclear risk of bias in several key domains including sampling, confounding and attrition in Bynum and adherence in Haines

^b^ Haines imprecision & variability in outcome in Bynum not reported

^c^ analyses consistent

^d^ Wide confidence intervals and low event rates

^e^ No serious concerns, some inconsistency in findings of analyses in Haines but credible explanation as contamination-adjusted ITT stronger than unadjusted or longer follow-up after time of intervention

Table S4 GRADE ratings of certainty of evidence: Primary Physician Visits to residents of aged care facilities during hospital admission (agreement of duplicate ratings by two independent reviewers)

| **Question: Does increased relational continuity of primary care through Primary Physician Visits during hospital admission improve outcomes and decrease resource use for residential aged care residents?**  Conclusion: The effect of Primary Physician Visits to residents of aged care facilities during hospital admission is uncertain (Very low certainty evidence) | | | | | | | |
| --- | --- | --- | --- | --- | --- | --- | --- |
| **Quality assessment**  **(observational evidence, commenced as low quality)** | | | | | | | **Summary of Findings** |
| **Studies**  **Participants (Follow up)** | **Risk of bias** | **Inconsistency** | **Indirectness** | **Imprecision** | **Other** | **Overall quality of evidence** | **Relative effect** (95% CI) |
|  |  |  |  |  |  |  |  |
| **Hospital admissions** | | | | | | | |
| 1 observational study (Susman, 1989),  N = 335  Mean LoS 9.6 - 13.1 days | No serious concerns | No serious concerns | No serious concerns | Serious concerns (-1) ^a^ | No serious concerns | VERY LOW  ⊕⊖⊖⊖ | Length of stay:  0 visits: 9.6 days; 1 visit: 11.4 days; 2 visits: 11.8 days; >3 visits: 13.1 days P<0.005 |
| **Function (ADLs)** | | | | | | | |
| 1 observational study (Susman, 1989),  N = 335  Mean LoS 9.6 - 13.1 days | No serious concerns | No serious concerns | No serious concerns | Serious concerns (-1) ^b^ | No serious concerns | VERY LOW  ⊕⊖⊖⊖ | A lower crude decrease in function score (-6.3 vs. -2.4, scale range 75 to -80, p<0.05  Number of physician visits was not associated with mean change in functional score. |
| **Mortality** | | | | | | | |
| 1 observational study (Susman, 1989),  N = 335  Mean LoS 9.6 - 13.1 days | No serious concerns | No serious concerns | No serious concerns | Serious concerns (-1) ^a^ | No serious concerns | VERY LOW  ⊕⊖⊖⊖ | The number of physician visits was not associated with mortality. |

Abbreviations: LoS, length of stay.

^a^ Total events <300
b <400 participants
